# UniBind: maps of high-confidence direct TF-DNA interactions across nine species

**DOI:** 10.1186/s12864-021-07760-6

**Published:** 2021-06-26

**Authors:** Rafael Riudavets Puig, Paul Boddie, Aziz Khan, Jaime Abraham Castro-Mondragon, Anthony Mathelier

**Affiliations:** 1 Centre for Molecular Medicine Norway (NCMM), Nordic EMBL Partnership, University of Oslo, 0349 Oslo, Norway; 2 Stanford Cancer Institute, Stanford University School of Medicine, Stanford, CA 94305 USA; 3 Department of Medical Genetics, Oslo University Hospital, Oslo, 0424 Norway

**Keywords:** Transcription factor binding sites, ChIP-seq, TF-DNA interactions, Transcription regulation, Evolutionary conservation, Cis-regulatory modules, UniBind

## Abstract

**Background:**

Transcription factors (TFs) bind specifically to TF binding sites (TFBSs) at cis-regulatory regions to control transcription. It is critical to locate these TF-DNA interactions to understand transcriptional regulation. Efforts to predict bona fide TFBSs benefit from the availability of experimental data mapping DNA binding regions of TFs (chromatin immunoprecipitation followed by sequencing - ChIP-seq).

**Results:**

In this study, we processed ~ 10,000 public ChIP-seq datasets from nine species to provide high-quality TFBS predictions. After quality control, it culminated with the prediction of ~ 56 million TFBSs with experimental and computational support for direct TF-DNA interactions for 644 TFs in > 1000 cell lines and tissues. These TFBSs were used to predict > 197,000 cis-regulatory modules representing clusters of binding events in the corresponding genomes. The high-quality of the TFBSs was reinforced by their evolutionary conservation, enrichment at active cis-regulatory regions, and capacity to predict combinatorial binding of TFs. Further, we confirmed that the cell type and tissue specificity of enhancer activity was correlated with the number of TFs with binding sites predicted in these regions. All the data is provided to the community through the UniBind database that can be accessed through its web-interface (https://unibind.uio.no/), a dedicated RESTful API, and as genomic tracks. Finally, we provide an enrichment tool, available as a web-service and an R package, for users to find TFs with enriched TFBSs in a set of provided genomic regions.

**Conclusions:**

UniBind is the first resource of its kind, providing the largest collection of high-confidence direct TF-DNA interactions in nine species.

**Supplementary Information:**

The online version contains supplementary material available at 10.1186/s12864-021-07760-6.

## Introduction

The regulation of gene expression is a complex process involving several biological mechanisms. The first step of the regulatory process controls where, when, and at which intensity RNAs are transcribed from their DNA template. This level of transcriptional regulation is mainly coordinated by transcription factors (TFs), which are DNA-binding proteins that recognize and bind short DNA sequences - their TF binding sites (TFBSs) [1]. TFs are known to co-operate through their combined binding at cis-regulatory regions proximal (promoters) or distal (enhancers or silencers) to the genes they regulate. These regions usually correspond to genomic locations locally dense in TFBSs, which are often referred to as cis-regulatory modules (CRMs), and act as genetic modulators to ensure appropriate gene expression [2].

The most popular experimental assay to detect TF-DNA interactions in vivo is chromatin immunoprecipitation followed by sequencing (ChIP-seq) [3]. After mapping the reads generated by ChIP-seq to the genome of interest, the computational analysis aims at identifying genomic regions enriched for mapped reads when compared to a control. The identified genomic locations are known as ChIP-seq peaks. TF ChIP-seq peaks usually span a few hundred base pairs. They derive from direct and indirect TF-DNA interactions [4], where the latter can emerge from protein-protein interactions between the ChIP’ed TF and another protein binding the DNA. Moreover, ChIP-seq peaks could also derive from non-specific binding of the TF to the DNA and noise/bias/artifacts. Several repositories store ChIP-seq peaks [5–9] and are freely available to the community. Nevertheless, these resources do not provide precise locations of the underlying direct TF-DNA interactions.

The TFBSs recognized by a TF are short (~ 10 bp-long) and degenerate sequences that can be modeled computationally for further predictions. The most widely used computational representations of TFBSs for a given TF are position weight matrices (PWMs), which summarize the probability of observing each nucleotide at each position within a set of observed TFBSs. Such computational models have recurrently been used to predict TFBSs in DNA sequences. For instance, one can apply PWMs to predict TFBSs in open chromatin regions (e.g. derived from DNase-seq or ATAC-seq [10–12]) or TF ChIP-seq peaks [13–15].

Previous efforts used PWMs to predict TFBSs within ChIP-seq peaks and made the predictions freely available [15–17]. These resources are specific to one or two species. A substantial limitation of the underlying computational approach is that it relies on the same pre-defined score threshold for all PWMs. Moreover, they do not fully exploit the ChIP-seq peak information such as the enrichment for the TF canonical binding motif close to the ChIP-seq peak summit - where most of the reads align [18]. To address these limitations, we recently developed the ChIP-eat software to specifically delineate direct TF-DNA interactions in ChIP-seq peaks and separate them from indirect or non-specific binding and ChIP-seq artifacts [14]. Briefly, ChIP-eat combines both computational (high PWM score) and experimental (centrality to ChIP-seq peak summit) support to find high-confidence direct TF-DNA interactions in a ChIP-seq experiment-specific manner. ChIP-eat was initially applied to 1983 ChIP-seq peak datasets for 232 human TFs to provide a map of direct TF-DNA interactions in the human genome, which contained > 8 million TFBSs stored in the UniBind database [14]. This collection of human TFBSs was proven useful to analyze cis-regulatory alterations in cancers [19–21] and other complex diseases [22, 23].

In this report, we describe the update of the UniBind database, which now stores > 72 million direct TF-DNA interactions predicted using an updated ChIP-eat pipeline on ~ 10,000 ChIP-seq peak datasets from nine species: *Arabidopsis thaliana*, *Caenorhabditis elegans*, *Danio rerio*, *Drosophila melanogaster*, *Homo sapiens*, *Mus musculus*, *Rattus norvegicus*, *Saccharomyces cerevisiae*, and *Schizosaccharomyces pombe*. After quality control, we provide the community with a robust collection of ~ 56 million TFBSs for 644 TFs in 1096 cell lines and tissues and > 197,000 cis-regulatory modules. A functional inspection of these TFBSs and CRMs highlighted that they are evolutionarily conserved and enriched at active cis-regulatory regions. Furthermore, we showed that this unique collection of TFBSs can predict TF binding combinatorics at cis-regulatory regions. Finally, we confirmed that a lower number of TFs binding at enhancers was associated with higher cell type and tissue specificity for these enhancers and vice-versa. The UniBind database is freely available online (https://unibind.uio.no/), through a programmatic RESTful API (https://unibind.uio.no/api/), and via genomic tracks (https://unibind.uio.no/genome-tracks/). Finally, it is accompanied with an enrichment tool to predict TFs with an enrichment of TFBSs in user-provided genomic regions (https://unibind.uio.no/enrichment/).

## Results

### Maps of direct TF-DNA interactions across nine species

#### Prediction of direct TF-DNA interactions

We aimed at providing a collection of direct TF-DNA interactions by combining experimental and computational approaches in several species. We applied an updated version of the ChIP-eat pipeline [14] to ChIP-seq datasets to discriminate high-confidence TFBSs within ChIP-seq peaks from indirect binding events and ChIP-seq noise/artifacts (see Methods). In a nutshell, ChIP-eat uses an entropy-based parameter-free algorithm to automatically define an enrichment zone, which contains TFBSs with high PWM scores and close proximity to ChIP-seq peak summits. These criteria provide computational (high PWM score) and experimental (proximity to peak summit) support for direct TF-DNA interactions. This process is carried out in a ChIP-seq dataset-specific manner. It first optimizes JASPAR PWMs [24] using the DAMO tool [25], which adjusts the PWMs through a perceptron algorithm to best discriminate ChIP-seq peaks from random sequences (see Methods). Next, the optimized PWMs are used to detect, for each dataset, the optimal thresholds on the PWM score and distance to the peak summits. These thresholds define the enrichment zone, which highlights direct TF-DNA interactions (see Methods and [14] for more details).

We collected ChIP-seq peaks for 11,373 ChIP-seq experiments from ReMap 2018 [26] and GTRD [5] for nine species: *Arabidopsis thaliana*, *Caenorhabditis elegans*, *Danio rerio*, *Drosophila melanogaster*, *Homo sapiens*, *Mus musculus*, *Rattus norvegicus*, *Saccharomyces cerevisiae,* and *Schizosaccharomyces pombe*. For 10,264 datasets, we were able to associate a TF binding profile in JASPAR with the ChIP’ed TF. The ChIP-eat pipeline was applied to each ChIP-seq peak dataset - JASPAR PWM pair independently to predict TFBSs. ChIP-eat identified enrichment zones to predict direct TF-DNA interactions in 9654 datasets. Altogether, this analysis culminated with the prediction of ~ 72 million TFBSs in ChIP-seq peaks for 841 TFs in 1316 cell lines and tissues (Supplementary Figure 1; Supplementary Table 1).

We provide these predictions through the UniBind database at https://unibind.uio.no/ (see section “UniBind web-application and web-services” for details). In the database, the datasets are annotated with information about the ChIP’ed TF (UniProt ID [27]), the cell line or tissue name with ontology IDs from Cellosaurus [28], Cell Line Ontology [29], Experimental Factor Ontology [30], UBERON [31], Cell Ontology [32], and BRENDA [33] whenever possible, and the treatment used, if any.

#### Quality control to establish a robust collection of direct TF-DNA interactions

In UniBind, we aimed to create a robust collection of bona fide direct TF-DNA interactions found in high-quality ChIP-seq peak datasets. This robust collection was obtained by implementing two quality control metrics and only retaining the datasets that satisfy the corresponding criteria. First, we expect high-quality ChIP-seq peak datasets to be enriched for the TF binding motif known to be bound by the ChIP’ed TF. Hence, we filtered out datasets where the DAMO-optimized TF binding motif, which maximizes the discrimination of ChIP-seq peaks from random sequences, was not similar to the expected canonical motif (see Methods). Second, we expect the ChIP-seq peaks to be enriched for TFBSs close to their summits. Hence, we filtered out the datasets where the predicted direct TF-DNA interactions did not show a significant enrichment around the summits (see Methods). While we provide the complete set of TFBSs predicted by ChIP-eat in the permissive collection to the community, we specifically contribute with the robust collection of quality-controlled direct TF-DNA interactions in high-quality ChIP-seq peak datasets.

**Fig. 1:**
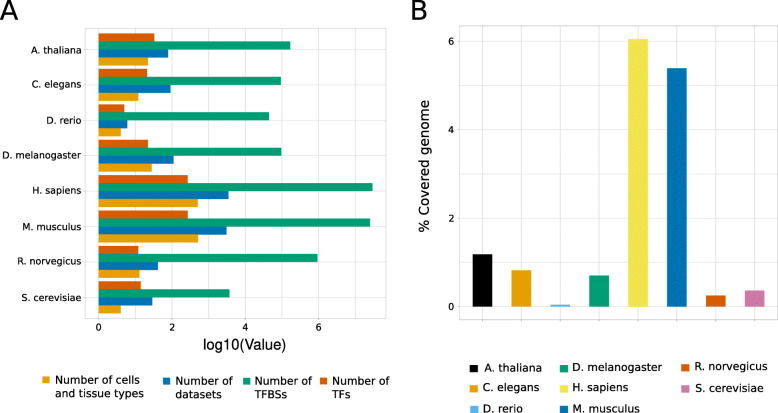
Overview of the UniBind robust collection. **A** Barplots showing the number of TFs (dark orange), TFBSs (green), datasets (blue), and cell and tissue types (light orange) stored in the robust collection of UniBind for each analyzed species. All values are log10-transformed. **B** Distribution of the percentages of the genomes covered by robust TFBSs in each species (one color per species, see legend)

After applying the quality-control filters, the robust collection of UniBind culminates with ~ 56 million TFBSs obtained from 6902 ChIP-seq peak datasets, all species combined (Fig. 1A; Supplementary Table 2). Note that none of the five datasets from *S. pombe* passed the quality-control criteria due to a lack of enrichment around the ChIP-seq peak summits. The TFBSs in the robust collection are associated with 644 distinct TFs ChIP’ed in 1096 cell lines and tissues (Fig. 1A; Supplementary Table 2). We found that the predicted TFBSs cover between 0.04 and 6.05% of the genome of their respective organism (Fig. 1B). For example, human and mouse TFBSs cover 6.05 and 5.39% of the genomes, respectively (Fig. 1B). Of course, these numbers are somehow a reflection of the number of ChIP-seq experiments available in the corresponding species (Supplementary Figure 2).

Since TFs are known to regulate transcription cooperatively through locally enriched TFBSs [2], we aimed to identify cis-regulatory modules (CRMs) corresponding to clusters of TFBSs. Specifically, we used CREAM [34] to locate DNA segments with local enrichment for UniBind TFBSs, which culminated with the predictions of > 197,000 CRMs (Supplementary Table 2).

With many TFs associated with multiple ChIP-seq datasets and similar TF binding profiles for TFs sharing DNA binding domains (DBDs) from the same structural class, the TFBS collection contains redundant instances. We aimed to reduce redundancy of the TFBS information to facilitate visualization, analyses, and interpretation [10]. Following the approach developed by Vierstra et al. [10], we defined TF binding archetypes representing similar TF binding profiles for TFs sharing DBD structural classes (see Methods). This approach allowed to identify a single TFBS location from several overlapping TFBSs predicted from TF profiles in the same archetype. Supplementary Figure 3 depicts a comparison between original and archetypal TFBSs at an exemplary genomic loci.

To summarize, we provide a collection of TFBSs with both experimental and computational support for direct TF-DNA interactions in quality-controlled ChIP-seq peak datasets. Hereafter, the complete collection of unfiltered TFBS predictions is referred to as the “permissive” collection, while the filtered, high-quality TF-DNA interactions are referred to as the “robust” collection.

### Support for the functional relevance of the TFBSs in the robust collection of UniBind

To further confirm the high-quality of the identified TFBSs in the robust collection of UniBind, we sought to provide support for their biological relevance. Hence, the analyses performed below were applied to the complete robust collection of TFBSs, except when explicitly stated otherwise.

#### Human and mouse TFBSs are evolutionarily conserved

**Fig. 2:**
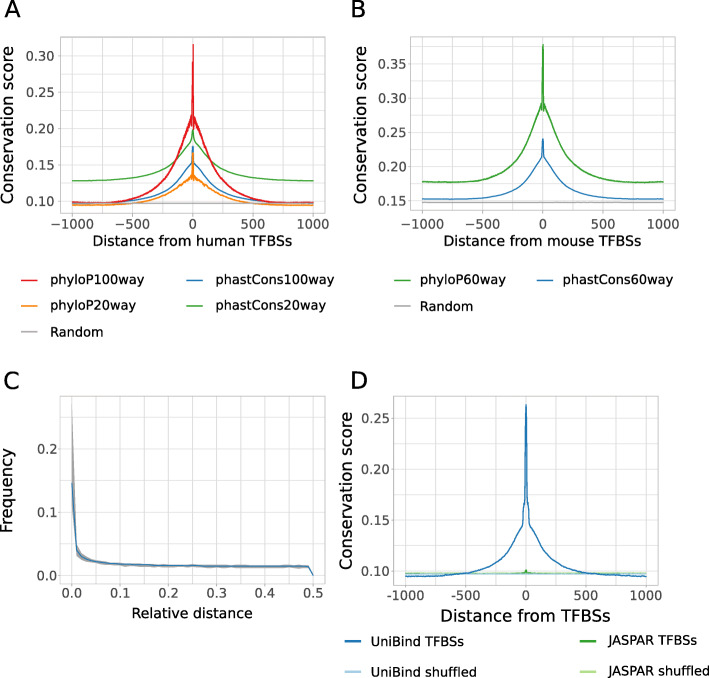
Evolutionary conservation of human and mouse TFBSs in the robust collection. Distributions of the average base-pair evolutionary conservation scores (phyloP and phastCons scores using multi-species genome alignments, see legends) at regions centered around human (**A**) and mouse (**B**) TFBSs from the robust collection. Random expectation (grey lines) was obtained by shuffling the original TFBS locations and obtaining the conservation score of the regions obtained. **C** Fraction of mouse lifted archetype TFBSs in the UniBind robust collection (y-axis) with respect to increasing relative distances (x-axis) from human archetype TFBSs from the same archetype computed using the *bedtools reldist* command. The figure provides, for each value of relative distance, the median (blue line) together with the 10th to 90th percentiles (grey area) of the observed frequencies. When two genomic tracks are not spatially related, one expects the fraction of relative distance distribution to be uniform. **D** Distributions of average base-pair evolutionary conservation scores (phastCons100way) at 1,000,000 randomly selected and shuffled TFBSs from JASPAR 2020 and UniBind 2021

We hypothesized that functionally relevant TFBSs should be enriched for evolutionary conservation. Indeed, conservation of DNA segments through evolution represents a hallmark of functional importance [35]. We considered evolutionary conservation scores in the human and mouse genomes computed by the PhyloP [36] and PhastCons [35] methods from the PHAST package [35]. Specifically, we investigated the average conservation of 2 kilobases (kb) DNA regions centered around the TFBS mid-points. Both scores estimate the probability of each nucleotide to belong to a conserved element [35, 36]. While phyloP scores reflect conservation of each nucleotide, phastCons scores consider flanking nucleotides to measure evolutionary acceleration (negative scores) and conservation (positive scores). For both human and mouse, we noticed that evolutionary conservation gradually increased when the distance to the TFBSs decreased, with sharp peaks of higher conservation at the TFBSs (Fig. 2). Increased evolutionary conservation was similarly observed at CRMs (Supplementary Figure 4). The signal was consistently found when considering multiple alignments of 19 (phyloP20way and phastCons20way, Fig. 2A) or 99 vertebrate genomes (phyloP100way and phastCons100way, Fig. 2A) to the human genome and 59 vertebrate genomes (phastCons60way and phyloP60way, Fig. 2B) to the mouse genome. The evolutionary conservation of TFBSs is not expected by chance as no conservation was observed when randomly shuffling the positions of the TFBSs in the human and mouse genomes (Fig. 2, grey lines). The acute increase of evolutionary conservation scores right at the TFBS locations reinforce the biological relevance of the direct TF-DNA interactions stored in UniBind.

To further investigate evolutionary conservation, we evaluated the conservation of predicted TFBSs at conserved elements between human and mouse. We lifted the mouse robust archetypal TFBSs over to the human genome and assessed their proximity to human TFBSs from the same archetype (see Methods). Next, we evaluated the relative distances between mouse archetypal TFBSs lifted over to the human genome and human TFBSs from the same archetype using *bedtools reldist* following [37, 38]. Across TF binding archetypes, we observed an enrichment for lifted mouse TFBSs to overlap human TFBSs (see the peak at distance 0, corresponding to an overlap, in Fig. 2C). The relative distances between mouse TFBSs lifted over to the human genome and human TFBSs confirm the enrichment for conservation of TFBSs between human and mouse associated with TFs sharing DBD structural classes.

Finally, we assessed the added value of the ChIP-eat approach to predict TFBSs over raw PWM mapping genome-wide. Specifically, we compared the evolutionary conservation of the TFBSs from the UniBind robust collection to TFBSs solely predicted from raw PWMs from JASPAR (see Methods). Supporting the functional relevance of UniBind TFBSs, we observed that UniBind TFBSs were significantly more evolutionarily conserved than TFBSs predicted from raw PWMs (Fig. 2D).

Altogether, these results pointed to the likely functional role of the TFBSs in transcriptional regulation of gene expression.

#### UniBind TFBSs are enriched at active promoters and enhancers

**Fig. 3:**
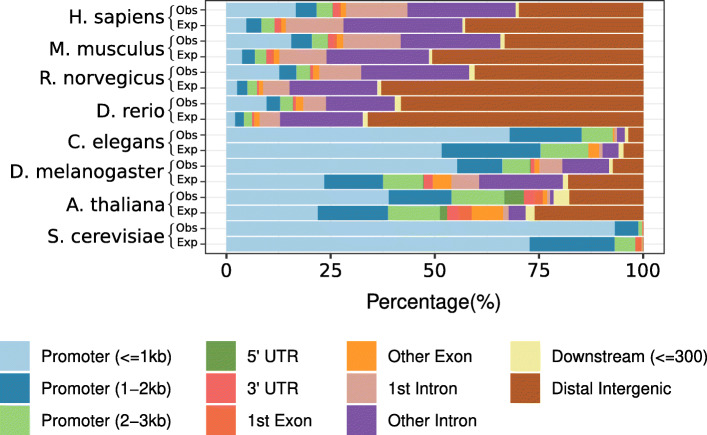
Genomic distribution of TFBSs. Distribution of the proportion of TFBSs from the robust collection overlapping with different types of genomic regions (columns; see legend) across species (rows). For each species, we provide the observed (first lines, denoted Obs) and expected (second lines, denoted Exp) proportions of TFBSs in each type of genomic regions. Expected proportions were estimated by randomly positioning the TFBSs in the corresponding genomes (see Methods)

Next, we sought support for the biological relevance of the TFBSs by assessing their overlap with cis-regulatory regions that are active in different cell types and tissues. We started by mapping out the distribution of the TFBSs with respect to promoter regions, 5′ and 3′ UTRs, exons, introns, regions downstream of genes, and distal intergenic regions (Fig. 3, top bar for each species). These distributions were compared to random expectations obtained by shuffling the TFBS positions along the corresponding genomes (Fig. 3, bottom bar for each species). By comparing the observed and expected distributions, we noticed that TFBSs were prominently found in promoter regions (< 1 kb upstream of transcription start sites). The enrichment for TFBSs in promoter regions was further confirmed by (i) OLOGRAM [39], which uses a Monte Carlo simulation approach and a negative binomial model to compute the significance of overlap between two sets of genomic regions (Supplementary Figures 5, 6, 7, 8, 9 and 10), and (ii) *bedtools reldist* [38], which computes the relative distances between the TFBSs and the genomic regions considered [37] (Supplementary Figure 11). Nevertheless, considering the distribution of TFBSs for each TF independently in each species revealed TFs with binding preferences for promoter regions while others prefer intronic or intergenic regions (Supplementary Figures 12, 13, 14, 15 and 16). The TSS-proximal versus TSS-distal preferences could explain the previously reported short- versus long-range regulatory effects of TFs [40].

**Fig. 4:**
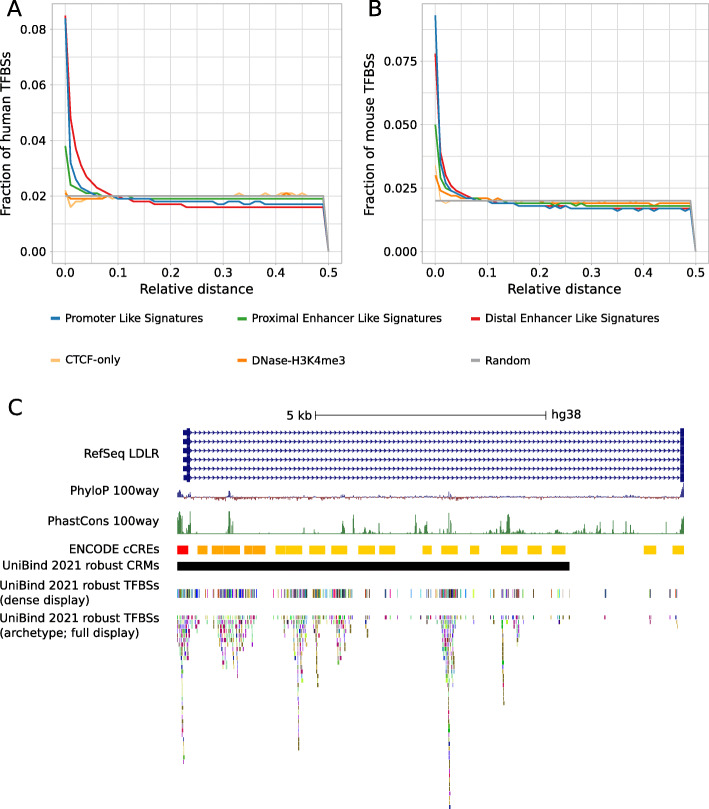
Analysis of the overlap of TFBSs with respect to active cis-regulatory regions in human and mouse. **A-B** Fraction of TFBSs in the UniBind robust collection (y-axis) with respect to increasing relative distances (x-axis) from ENCODE candidate cis-regulatory regions (cCREs) computed using the *bedtools reldist* command for human (A) and mouse (B). When two genomic tracks are not spatially related, one expects the fraction of relative distance distribution to be uniform. **C** Genomic tracks from the UCSC Genome Browser at the human LDLR gene locus (from start to first coding exon) providing information about PhyloP and PhastCons evolutionary conservation scores and the locations of ENCODE cCREs, UniBind CRMs, UniBind TFBSs from the robust collection (using the dense display mode to maximally condense the track) and the non-redundant collection of archetype TFBSs. Colors in the ENCODE cCREs track indicate: promoter-like signature (red), proximal enhancer-like signature (orange), and distal enhancer-like signature (yellow)

In the vertebrate species (human, mouse, rat, and zebrafish), the majority of TFBSs lie in introns and distal intergenic regions (Fig. 3), which is expected given the large portion of the corresponding genomes covered by these non-coding regions. To confirm the biological function of the TFBSs stored in UniBind, we examined their overlap with active cis-regulatory regions in the mouse and human genomes. We considered the candidate cis-regulatory elements (cCREs) predicted using epigenetic marks by the ENCODE consortium [41]. Specifically, DNase I hypersensitive open chromatin regions were first identified and then overlapped with H3K4me3 and H3K27ac histone modification marks and CTCF ChIP-seq data to predict five types of cCREs with: (1) a promoter-like signature (PLS), (2) an enhancer-like signature proximal (pELS) or (3) distal (dELS) to TSSs, (4) a H3K4me3 signature (DNase-H3K4me3), or (5) a CTCF-only signature [41]. Consistently, we confirmed that UniBind TFBSs were enriched in PLS and ELS cCREs when considering both the OLOGRAM and *bedtools reldist* evaluations of overlap (Fig. 4A-B; Supplementary Figures 17, 18). The enrichment at regions of active promoter signature is consistent with the genomic distribution observed above. The enrichment at regions harbouring active enhancer signature suggests that the TFBSs are not randomly spaced in the introns and intergenic regions. Furthermore, we confirmed that CRMs were enriched for cCREs with active promoter- or enhancer-like signatures when considering the 105,104 and 73,917 CRMs predicted in human and mouse, respectively (Supplementary Figures 19, 20 and 21). Figure 4C shows an example of the UCSC Genome Browser [42] at the human LDLR gene locus where we observe the overlap between UniBind TFBSs, CRMs, and cCREs.

Together, these results highlight the biological relevance of the UniBind TFBSs and CRMs for transcriptional regulation via their association with active promoters and enhancers in human and mouse.

### Specificity of enhancer activity in cell types and tissues correlates with binding TF composition

**Fig. 5:**
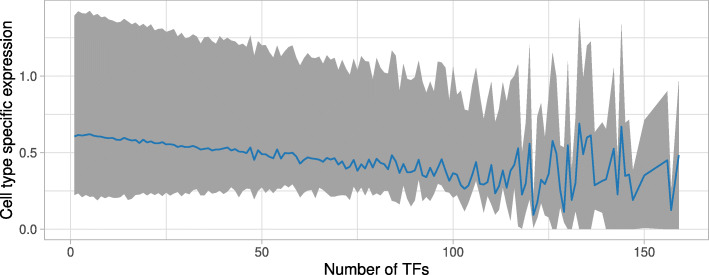
Correlation between enhancer activity and TF binding. For each enhancer predicted using Cap Analysis of Gene Expression (CAGE) by the FANTOM5 consortium, we computed the number of TFs with overlapping TFBSs in the robust collection of UniBind (x-axis). The figure provides, for each value of the number of TFs found to bind in enhancers, the median (blue line) together with the 10th to 90th percentiles (grey area) of cell type specific activity of these enhancers. The expression measures were derived from CAGE (capturing enhancer RNA expression). The specificity of activity (y-axis) is provided within the [0; 1] range with 0 representing ubiquitous enhancer activity and 1 exclusive expression activity

We further investigated how the number of TF binding events at enhancers could be related to their regulatory effects. We considered enhancers that were identified through the capture of bidirectional transcription of enhancer RNAs (eRNAs) at their boundaries using Cap Analysis of Gene Expression (CAGE) in 1829 human libraries [43]. Cell type and tissue specificity was assessed by considering the amount of eRNAs captured by CAGE across the libraries [43]. We overlapped the UniBind TFBSs with the CAGE-derived enhancers and assessed the relationship between the expression specificity of the enhancers and the number of TFs with binding sites in these enhancers. We observed that cell type / tissue specific enhancers tend to harbour a lower number binding TFs, while more ubiquitously active enhancers tend to harbour a higher number of binding TFs (Fig. 5; Supplementary Figure 22). The correlation between the number of binding TFs and cell type / tissue expression specificity of enhancers is in line with previous observations showing an association between the number of TFBSs and the combinatorics of TFs at promoters and enhancers with enhancer activity strength and specificity [44–46]. Altogether, these observations underline the importance of TF cooperation for cis-regulatory activity.

### UniBind TFBSs reveal TF binding combinatorics at cis-regulatory regions

**Fig. 6:**
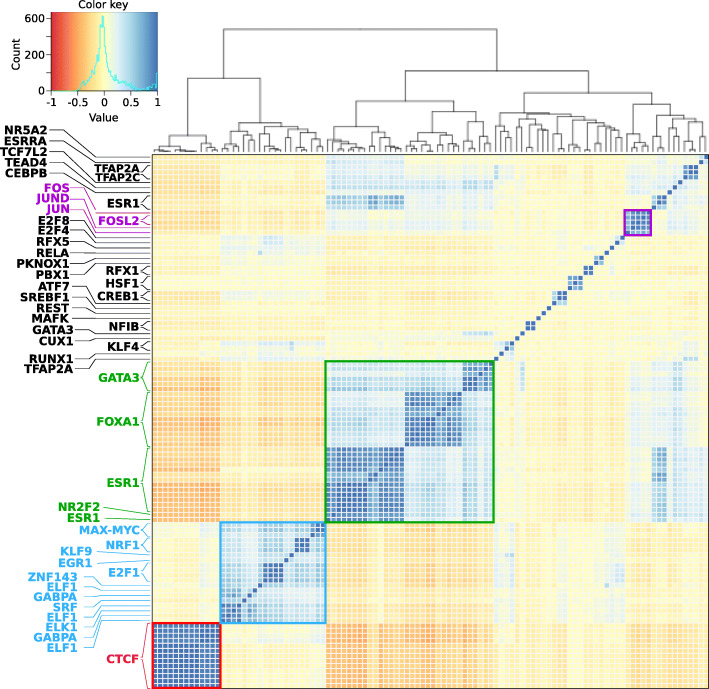
TF combinatorial binding in invasive breast ductal carcinoma. Hierarchical clustering of the pairwise Pearson correlation coefficient between all TFBSs from untreated MCF7 cells from the robust collection of UniBind. Different clusters and their respective TFs are coloured in red, blue, green, and purple. In the heatmap, blue colors indicate a higher positive correlation coefficient between datasets, while red colors indicate an anticorrelation (see legend)

We explored the capacity of UniBind TFBSs to further pinpoint relevant TF binding combinatorics at cis-regulatory regions. As a case study, we examined the direct TF-DNA interactions stored in UniBind and derived from ChIP-seq experiments in the untreated MCF7 cell line. This cell line is representative of estrogen receptor positive (ER+) invasive ductal breast carcinoma, which is known to be mainly driven by the combined activity of the TFs ESR1, GATA3, and FOXA1 [47]. We extended the genomic locations of UniBind TFBSs predicted in MCF7 by 50 bp on each side and intersected these regions between each pair of MCF7 TFBS datasets using the Intervene tool [48]. Next, we computed the fractions of overlap for each pair and calculated the pairwise Pearson correlation coefficients of the fractions of overlap between all pairs of datasets. A high pairwise correlation coefficient between two datasets indicates that the underlying TFBS regions are co-localizing. Hierarchical clustering of the pairwise correlation coefficient revealed 4 main clusters (Fig. 6). As expected, we observed high correlations between datasets for the same TF (e.g. red cluster in Fig. 6 with exclusively CTCF TFBSs). The largest cluster (Fig. 6, green) was mainly composed of TFBSs from ESR1, FOXA1, and GATA3. Co-localization of binding events for these TFs confirm the potential of UniBind TFBSs to highlight TFs known to cooperate at cis-regulatory regions. The second largest cluster (Fig. 6, blue) contained TFBSs for E2F1, NRF1, MAX, MYC, ELK1, ELF1, GABPA, EGR1, and SRF. Among these TFs, MAX and MYC as well as ELK1 and SRF are known to dimerize to bind DNA. Finally, the purple cluster was composed of JUN and FOS TFBSs, known to bind DNA as a dimer to form the AP1 complex. This case study exemplifies how UniBind TFBSs can be used to derive biologically relevant information about TF binding combinatorics.

Altogether, the assessments of the functional and biological relevance to study transcriptional regulation outlined here support, a posteriori, the high-quality of the direct TF-DNA interactions stored in the robust collection of UniBind.

### UniBind web-application and web-services

#### Accessing and exploring UniBind data

All the direct TF-DNA interactions from the permissive and robust collections are freely available through the UniBind web-application at https://unibind.uio.no. The predictions come with metadata about the associated ChIP-seq experiments and external links to useful resources such as ReMap [26], GeneCards [49], and GEO [50]. Users can search and explore the data through the user-friendly web-interface. The web-application provides a search interface for users to filter the datasets using the metadata fields and search results are downloadable as a metadata table as well as FASTA and BED files for the TFBSs. To improve the searchability of the data, the search engine supports gene synonyms when searching for TFs. All data can be downloaded for individual datasets as well as through bulk download links per species or collection. In addition, we developed a RESTful API (https://unibind.uio.no/api/) to allow programmatic access to the stored data from any programming language. Finally, we built genome track hubs that are easily visualized through the UCSC [51] and Ensembl [52] genome browsers. The track hubs can be accessed through the UniBind web-application (https://unibind.uio.no/genome-tracks/) as well as through the public track hubs at UCSC [51] and the track hub registry (https://trackhubregistry.org/).

#### TFBS sets enrichment application tool

**Fig. 7:**
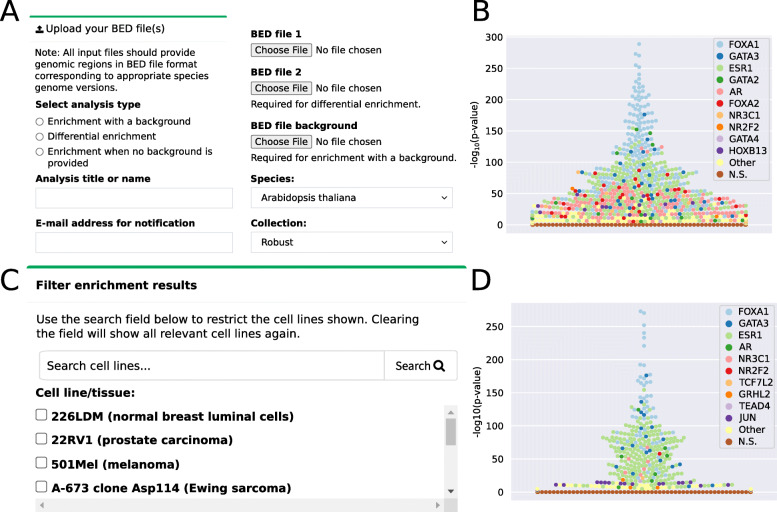
The UniBind TFBS set enrichment tool. **A** The UniBind enrichment web-application allows users to select the enrichment analysis type, set a title, provide an email address for notification upon completion of the analysis, upload of the required input files based on the enrichment analysis type, and select the species and collection to compute the enrichment. **B** Enrichment results shown as swarm plots of the -log_10_(*p*-values) (Fisher exact tests; see Methods). Each point corresponds to a TFBS set for a given TF in a given ChIP-seq experiment. Distinct colors are assigned to the top 10 TFs with at least one TFBS set enriched (see legend). **C** The enrichment results can be further explored by restricting the output to TFBS sets obtained in specific cell lines and tissues, which can be searched by keywords and selected. **D** Swarm plot similar to (**B**) but restricted to TFBS sets obtained from breast-related tissues and cell lines

A regular task when studying transcriptional regulation is to find TFs that are the most likely to control the activity of a set of cis-regulatory regions. Classical strategies rely on the prediction of enriched potential TFBSs for a set of TFs derived from either ChIP-seq peaks datasets [53–56] or PWM predictions [55, 57]. As UniBind stores TFBSs with both ChIP-seq and PWM evidence of direct TF-DNA interactions, one can rely on this resource to infer the TFs likely to bind a set of cis-regulatory regions. The method consists in computing the enrichment for specific TFBS sets in given DNA regions compared to background regions. We provide a web-service (and the underlying source code) to perform this TFBS dataset enrichment analysis to the users at https://unibind.uio.no/enrichment/ (Fig. 7A). The enrichment computation relies on the Locus Overlap Analysis (LOLA) tool [58]. The enrichment tool provides three different types of enrichment analyses: (1) using a provided universe of potentially bound regions; (2) comparing enrichment with another set of genomic regions to perform differential enrichment; or (3) comparing the enrichment to all TFBSs stored in UniBind as a universe (Fig. 7A).

As a case example, we applied the enrichment tool to genomic regions surrounding CpGs found to be demethylated in ER+ breast cancer patients [59]. As a background set, we used all CpG probes from the Illumina Infinium HumanMethylation450 microarray. The demethylated CpG regions in ER+ patients were predicted to be bound by FOXA1, GATA2, GATA3, ESR1 and AR (top 5 TFs, Fig. 7B). The enrichment of these TFs is in line with the known ER+ TF drivers [59]. Further, the enrichment tool allows users to filter the results by restricting the search to TFBS datasets derived from specific cell lines / tissues. In our case study, limiting to breast-related cell types and tissues highlights FOXA1, GATA3, and ESR1 with the most enriched TFBS sets (Fig. 7C-D), which is in agreement with the driving role of these TFs in ER+ carcinogenesis.

## Discussion

Through the uniform processing of > 10,000 ChIP-seq peak datasets, we provide maps of direct TF-DNA interactions in nine species. Altogether, this process culminated with the prediction of > 72 million TFBSs, ~ 56 million of which passed stringent QC criteria to compose the robust collection of direct TF-DNA interactions in UniBind. The robust collection is associated with 644 distinct TFs from 6902 ChIP-seq datasets derived from 1096 cell lines and tissues. Functional assessments of the robust collection of TFBSs through evolutionary conservation and strong overlap with active promoters and enhancers in human and mouse highlighted the high-quality and biological relevance of the collection. Further, we showed that the TFBSs can provide insights into enhancer activity and TF binding combinatorics at cis-regulatory regions. Previous works combined with the results outlined here underline the functional relevance of analyzing TFBS combinatorics at cis-regulatory elements to shed light on the molecular mechanisms underlying transcriptional regulation. Besides the functional assessment done in this manuscript, we showed in the original publication describing ChIP-eat [14] that predicted TFBSs likely represent direct TF-DNA interactions, which are recapitulated by the ChExMix complementary approach on ChIP-exo [60]. While the predictions show strong experimental and computational support for their biological relevance, it is expected that not all TFBSs and CRMs stored in UniBind have a biological function. However, the predictions provide the community with an unprecedented access to high-quality TFBSs across nine species.

We provide this resource freely to the community through a dedicated web-application, a RESTful API, and genome tracks for the UCSC and Ensembl genome browsers. Finally, TFBS dataset enrichment analyses can be performed through an online web-service and a stand-alone tool to predict the TFs acting upon a set of genomic regions.

The TFBS predictions provided in the current version of UniBind were obtained using PWMs as computational models. While several resources storing TF PWMs exist, we decided to rely on high-quality JASPAR profiles from the CORE collection, which have been manually curated and confirmed via orthogonal evidence. In the original version of UniBind dedicated to human [14], we provided predictions obtained from four different computational models: PWMs, binding energy models [61], transcription factor flexible models (TFFMs) [62], and DNA shape-based models (DNAshapedTFBS models) [63]. The ChIP-eat pipeline is agnostic to the computational model used to predict the enrichment zone with high computational and experimental evidence of direct TF-DNA interactions. Hence, we foresee that more sophisticated models than PWMs could be used to predict TFBSs to be stored in UniBind in the future, should they become extensively used by the community.

UniBind relies on the availability of ChIP-seq peak datasets made available to the community. The current release relies on the ReMap and GTRD databases. These databases were selected as they (1) encompass a large part of the publicly available ChIP-seq experiments for several species, (2) process ChIP-seq data uniformly, and (3) are regularly updated and under active maintenance. UniBind will be updated on a regular basis, as soon as new ChIP-seq datasets become available in ReMap and GTRD. Moreover, we are open to including other ChIP-seq peak resources that fulfill the criteria described above (e.g. repositories specialized in some species or taxa) for the upcoming updates of UniBind.

## Methods

### ChIP-seq peak datasets and TF binding profiles

A total of 11,373 ChIP-based datasets with peaks predicted by MACS [64] were retrieved from the ReMap (2018 version) [26] and GTRD [5] databases. ReMap datasets were the same as the ones used in the previous UniBind release and were reprocessed with new JASPAR PWMs. Note that some datasets were obtained using the ChIP-seq or ChIP-exo protocols; we refer to ChIP-seq datasets as a whole in this manuscript for simplicity.

ChIP-seq peak datasets were associated with JASPAR (version 2020) [24] TF binding profiles (provided as position frequency matrices, PFMs) whenever possible. Specifically, we used the HGNC gene symbols to search the collection of JASPAR TF binding profiles in the same taxonomic group as the ChIP’ed TF. For the datasets where no TF binding profile was found, we used the *mygene* bioconductor package [65] to obtain all possible gene synonyms and used the synonyms to search for JASPAR TF binding profiles. We filtered out ChIP-seq datasets for which no JASPAR PFM was found for the ChIP’ed TF. Altogether, JASPAR PFMs were assigned to 10,264 datasets out of 11,373. Note that some ChIP-seq peak datasets stored in ReMap and GTRD are not associated with TFs but general transcriptional regulators (e.g. EP300, RAD21, SMC4), so no PFM in JASPAR could be assigned; for some TFs, no PFM was available in JASPAR.

### Genome assemblies

The genome assemblies used for each species were: *hg38* (*H. sapiens*), *mm10* (*M. musculus*), *Rnor_6.0* (*R. norvegicus*), *WBcel235* (*C. elegans*), *dm6* (*D. melanogaster*), *GRCz11* (*D. rerio*), *TAIR10* (*A. thaliana*), *R64–1-1* (*S. cerevisiae*), and *ASM294v2* (*S. pombe).*

### Identification of direct TF-DNA interactions

We applied the ChIP-eat pipeline (https://bitbucket.org/CBGR/chip-eat/) to each ChIP-seq peak dataset independently, following a similar method to the one described in [14]. Compared to the original version of ChIP-eat [14], we made the two following modifications: (1) we used DAMO [25] (version 1.0.1) with default parameters to optimize the PWMs in a dataset-specific manner; (2) once the thresholds (on the distance to peak summits and PWM score) defining the enrichment zone were predicted, we rescanned the peaks with the DAMO-optimized PWMs and kept the best hit (highest PWM score) per peak that fall within the enrichment zone, if any. DAMO was used to optimize the JASPAR PWMs in a ChIP-seq dataset-specific manner following the approach described in [14]. Specifically, for each ChIP-seq dataset, we considered (i) sequences of ∓50 bp around the ChIP-seq peak summits as a positive set and (ii) 100 bp genomic sequences matching the %GC content of the positive sequences using the *g* subcommand of BiasAway [66]. DAMO used a perceptron training strategy to find the optimal PWM that maximizes the area under the receiver operating curve, which evaluates the discriminative power of a PWM between sequences from the positive and negative sets [25].

### Quality control metrics for the robust collection

Quality control was performed on all processed datasets. TFBSs in the permissive collection were filtered using two quality control (QC) metrics. (1) To ensure similarity between the DAMO-optimized PFM and the original JASPAR PFM, we only kept in the robust collection the datasets providing a TOMTOM (version 4.11.4) [67] similarity *p*-value strictly below 0.05. This QC metric ensures that the canonical motif known to be recognized by the ChIP’ed TF is enriched in the ChIP-seq peaks. (2) To ensure a strong enrichment for direct TF-DNA interactions in the vicinity of the peak summits, we computed a centrality enrichment following the method described in CentriMo [68]. Only TFBS datasets with a centrality *p*-value < 0.05 were kept in the robust collection. This QC metric ensures that TFBSs are enriched in the vicinity of the peak summits overall in the ChIP-seq peaks considered (some of which are not predicted to contain a direct TF-DNA interactions / TFBS).

### Computation of descriptive statistics

For both the robust and permissive collections, the number of TFBSs was computed as the sum of the number of unique instances of genomic loci bound by each TF. The computation was performed by extracting the columns of interest from the BED files for an organism and collection, sorting them using the *sort -k1,1 -k2,2n* command and getting the unique instances using the *uniq* command. Finally, the number of unique instances were counted using the *wc -l* command.

Proportions of the covered genome were computed by dividing the total number of nucleotides covered by the TFBSs by the total number of nucleotides in the genome. To compute the number of nucleotides covered by the TFBSs, we compiled a BED file for all TFBSs, sorted the genomic regions using the *sort -k1,1 -k2,2n* command, and subsequently merged the overlapping locations using the *merge* subcommand from *bedtools* (version 2.26.0).

### TF motif archetypes and archetypal TFBSs

TF motif archetypes were computed following the approach described in [10]. We retrieved PFMs from JASPAR 2020 [24] for insects, fungi, nematodes, plants, and vertebrates. For each taxon, we computed pairwise similarity between all PFMs using Tomtom [67]. The e-values computed by Tomtom were -log_10_ transformed. The corresponding values were used to perform hierarchical clustering of the PFMs using correlation distance as the distance metric and complete linkage as the clustering method with the *cluster* library from scipy (version 1.3.0). Next, we manually inspected the hierarchical clusterization to define clusters of similar PFMs. For each cluster, we computed the archetype motif associated to each DBD structural class by aligning all PFMs and creating a consensus motif following the method used in [10] (code available at https://bitbucket.org/CBGR/unibind_manuscript/).

### Cis-regulatory modules

For each species, we considered unique locations of permissive and robust TFBSs separately and used CREAM [34] with default parameters to compute cis-regulatory modules.

### Random positioning of TFBSs

The random distribution of TFBSs was obtained by shuffling the original unique TFBS coordinates along the genomes using the *shuffle* subcommand of the *bedtools* (version 2.25.0) [38] with the *-chrom* option to keep the same number of TFBSs per chromosome.

### Evolutionary conservation

The evolutionary conservation scores were retrieved from the UCSC genome browser data portal as bigWig files for the human and mouse genomes. Specifically, we downloaded the bigWig files corresponding to the tracks phastCons100way, phastCons20way, phyloP100way, and phyloP20way for human and phastCons60way and phyloP60way for mouse. We considered unique locations of human and mouse TFBSs from the robust collection and the average conservation scores in 2 kb regions centered around the TFBS mid-points were computed using the *agg* subcommand of *bwtool* (version 1.0) [69]. The same strategy was applied to the random positions of TFBSs and the CRMs.

We retrieved genome-wide JASPAR TFBSs predicted from raw PWMs (at http://expdata.cmmt.ubc.ca/JASPAR/downloads/UCSC_tracks/2020/hg38/) for all TF binding profiles associated with the UniBind robust TFBS collection. We randomly sampled 1,000,000 TFBSs ten times from JASPAR and UniBind TFBSs, respectively. For each set of randomly selected TFBSs, we computed the average evolutionary conservation in the surrounding genomic regions following the methodology described above. Moreover, for each iteration, we shuffled the TFBSs with the subcommand *shuffle* from bedtools (version 2.26.0) to compute the random expectation of evolutionary conservation scores. Finally, we plotted, in Fig. 2D, the median conservation score over the 10 random sampling for the UniBind and JASPAR predicted/shuffled TFBSs.

### Genomic distributions

For each species, the genomic coordinates of all TFBSs were retrieved and duplicate coordinates (from multiple ChIP-seq experiments) were filtered out to conserve only unique genomic locations. The distributions of these unique TFBS positions with respect to promoters, 5′ and 3′ UTRs, exons, introns, regions downstream of genes, and intergenic regions were obtained using the ChIPseeker Bioconductor package (version 1.20.0) [70]. We used the following genome annotations with ChIPseeker: TxDb.Athaliana.BioMart.plantsmart28 (*A. thaliana)*, TxDb.Celegans.UCSC.ce11.refGene (*C. elegans*), TxDb.Drerio.UCSC.danRer11.refGene (*D. rerio*), TxDb.Dmelanogaster.UCSC.dm6.ensGene (*D. melanogaster*), TxDb.Hsapiens.UCSC.hg38.knownGene (*H. sapiens*), and TxDb.Mmusculus.UCSC.mm10.knownGene (*M. musculus*). The genome annotations for *R. norvegicus* and *S. cerevisiae* were built from GTF files obtained from Ensembl by using the *makeTxDbFromGFF* function from the *GenomicFeatures* Bioconductor package [71] (version 1.36.4). The same methodology was applied to the random distribution of TFBSs.

The enrichment for the unique TFBS positions at the different genomic features was computed using the OLOGRAM function of the *gtftk* package (version 1.2.1) [39, 72]. Note that no result is provided for *H. sapiens* and *M. musculus* as OLOGRAM did not manage to complete the computations.

### Relative distances and enrichment with candidate cis-regulatory elements (cCREs)

The genomic coordinates of human and mouse cCREs predicted by ENCODE were retrieved as BED files from the SCREEN web-portal at https://screen.encodeproject.org/.

The relative distances between the unique TFBS positions and the ENCODE cCREs were computed using the *reldist* subcommand of the *bedtools* (version 2.25.0). The same methodology was applied to the CRMs and the randomly distributed TFBSs.

The enrichment for the unique TFBS positions at the ENCODE cCREs was computed using the OLOGRAM function of the *gtftk* package (version 1.2.1) [39, 72]. The same methodology was applied to the CRMs.

### Cell type and tissue specific enhancer expression

The genomic coordinates (hg19 genome assembly) of the 43,011 permissive enhancers predicted from CAGE experiments [43] were retrieved as BED files from http://enhancer.binf.ku.dk/presets/. Coordinates were converted to the hg38 genome assembly using the UCSC *liftOver* tool [73]. For each TF, we considered unique genomic coordinates and intersected these locations with the enhancer coordinates using the *intersect* subcommand of the *bedtools* (version 2.29.2) using the options *-wa -filenames -C*. The results were used to compute the number of TFs with at least one TFBS overlapping the enhancers.

Enhancer cell type and tissue specific expressions were obtained from Andersson et al. [43] and computed as $1-\left(\frac{entropy(enhancerexpression)}{\log 2\left( numberofcelltypes/ tissues\right)}\right)$. The vector of expression values for each enhancer over cell types or tissues corresponded to the mean of the enhancer expression in each cell type or tissue [43].

### Pairwise correlation computation for TFBS datasets from MCF7

The TFBS datasets associated with the MCF7 cell line were retrieved from the UniBind database using the search functionality of the web-application. Metadata was used to restrict the datasets to the ones where no treatment was introduced in the MCF7 cells. For each dataset, TFBS positions were expanded by 50 bp on each side using the *slop* subcommand of the *bedtools* and then merged using the *sort* and *merge* subcommands of the *bedtools*. These genomic regions were used as input to the *pairwise* subcommand of the Intervene tool [48] to compute the fraction of intersections between each pair of datasets. Pairwise Pearson correlation coefficients between the vectors of fraction of intersections between each pair of datasets were computed using Intervene. Hierarchical clustering was obtained through the Intervene Shiny application (https://intervene.shinyapps.io/intervene/) with the *Heatmap.2* function.

### Genome track hubs

Genome track hubs were built following the specifications at https://genome.ucsc.edu/goldenPath/help/hgTrackHubHelp.html. Moreover, we computed the “archetype” track for the robust collection with non-redundant binding events (see section TF motif archetypes and archetype TFBSs).

### Enrichment tool and web-service

The enrichment tool relies on the LOLA Bioconductor package (version 1.14.0) [58] to assess enrichment of overlaps based on Fisher exact tests. For each species, a dedicated LOLA database was built with all the predicted TFBSs and the corresponding metadata informing about cell type / tissue, treatment, and TF name. The databases were generated following the instructions provided at http://databio.org/regiondb and are available as RDS R objects on Zenodo at 10.5281/zenodo.4704641. The web-service is freely available at https://unibind.uio.no/enrichment/ with source code for the standalone software available at https://bitbucket.org/CBGR/unibind_enrichment/.

### UniBind web-application

The UniBind web-application is developed in Python using the model-view-controller framework Django. It uses SQLite to store TFBS metadata and Bootstrap as the frontend template engine. The search function relies on the RESTful API (see below). It allows for searching for gene name synonyms using naming data from the Entrez Gene and SwissProt databases and combining such data with JASPAR matrix profile information to yield a relevant collection of synonyms (source code at https://bitbucket.org/CBGR/synonyms). The source code of the UniBind web-application together with installation instructions are available at https://bitbucket.org/CBGR/unibind.

### RESTful API

The RESTful API is implemented in Python as part of the UniBind web-application using the Django REST Framework. An Apache HTTP server provides access to the application and thus to the API, with the underlying SQLite database system supporting queries constructed by the API implementation to retrieve data requested by users of the API. The available REST API endpoints are “Datasets”, “Cell types”, “Collections”, “Species”, and “Transcription factors”. The API is available at https://unibind.uio.no/api/.

## Supplementary Information

**Additional file 1: Table S1.** Overview of the permissive collection. Table providing the number of datasets, TFs, cell / tissue types, and TFBSs in the permissive collection of UniBind. The number of TFBSs was computed as the number of unique instances of genomic loci bound by a TF. **Table S2.** Overview of the robust collection. Table providing the number of datasets, TFs, cell /tissue types, and TFBSs in the robust collection of UniBind. The number of TFBSs was computed as the number of unique instances of genomic loci bound by a TF. **Figure S1.** Visual overview of the permissive collection. (**A**) Barplots showing the number of TFs (dark orange), TFBSs (green), datasets (blue), and cell and tissue types (light orange) stored in the permissive collection of UniBind for each analyzed species. All values are log10-transformed. (**B**) Distribution of the percentages of the genomes covered by robust TFBSs in each species (one color per species, see legend). **Figure S2.** Relationship between number of datasets and genome coverage. Scatter plots representing the percentage of genome coverage (y-axes) with respect to the number of datasets in the permissive (**A**) and robust (**C**) collections or the number of TFs in the permissive (**B**) and robust (**D**) collection (x-axes). Each colored point in each panel represents the data associated to one species (see legend for color coding). **Figure S3.** The UniBind 2021 compressed and robust tracks with all TFBSs from the robust human collection. An example of a random genomic locus showing the comparison between the original and archetypal TFBSs. The tracks shown are, from top to bottom: RefSeq track with the first intron of the human TTC6 gene, the UniBind compressed track with archetypal TFBSs, and the UniBind robust track showing all TFBSs at the same location. **Figure S4.** Evolutionary conservation at human and mouse robust CRMs. Distributions of the average base-pair evolutionary conservation scores (phyloP and phastCons scores using multi-species genome alignments, see legend) at regions centered around UniBind human (**A**) and mouse (**B**) CRMs from the robust collection. Conservation of random CRMs was obtained by shuffling the original CRMs and obtaining the conservation score of the new regions. **Figure S5.** Enrichment analysis for *A. thaliana* TFBSs in genomic regions. Barplots representing the expected (grey bars) versus observed (blue bars) overlap lengths (**A**) or number of intersections (**B**) between *A. thaliana* TFBSs from the robust collection and genomic annotations (x-axis). The plots and computed *p*-values (green: enrichment; orange: depletion) were obtained using the OLOGRAM command of the GTF toolkit. **Figure S6.** Enrichment analysis for *C. elegans* TFBSs in genomic regions. Barplots representing the expected (grey bars) versus observed (blue bars) overlap lengths (**A**) or number of intersections (**B**) between *C. elegans* TFBSs from the robust collection and genomic annotations (x-axis). The plots and computed p-values (green: enrichment; orange: depletion) were obtained using the OLOGRAM command of the GTF toolkit. **Figure S7.** Enrichment analysis for *D. rerio* TFBSs in genomic regions. Barplots representing the expected (grey bars) versus observed (blue bars) overlap lengths (**A**) or number of intersections (**B**) between *D. rerio* TFBSs from the robust collection and genomic annotations (x-axis). The plots and computed p-values (green: enrichment; orange: depletion) were obtained using the OLOGRAM command of the GTF toolkit. **Figure S8.** Enrichment analysis for *D. melanogaster* TFBSs in genomic regions. Barplots representing the expected (grey bars) versus observed (blue bars) overlap lengths (**A**) or number of intersections (**B**) between *D. melanogaster* TFBSs from the robust collection and genomic annotations (x-axis). The plots and computed p-values (green: enrichment; orange: depletion) were obtained using the OLOGRAM command of the GTF toolkit. **Figure S9.** Enrichment analysis for *R. norvegicus* TFBSs in genomic regions. Barplots representing the expected (grey bars) versus observed (blue bars) overlap lengths (**A**) or number of intersections (**B**) between *R. norvegicus* TFBSs from the robust collection and genomic annotations (x-axis). The plots and computed p-values (green: enrichment; orange: depletion) were obtained using the OLOGRAM command of the GTF toolkit. **Figure S10.** Enrichment analysis for *S. cerevisae* TFBSs in genomic regions. Barplots representing the expected (grey bars) versus observed (blue bars) overlap lengths (**A**) or number of intersections (**B**) between *S. cerevisae* TFBSs from the robust collection and genomic annotations (x-axis). The plots and computed p-values (green: enrichment; orange: depletion) were obtained using the OLOGRAM command of the GTF toolkit. **Figure S11.** Analysis of the overlap of robust TFBSs with respect to genomic annotations in all species in UniBind. Fraction of TFBSs in the UniBind robust collection (y-axis) with respect to increasing relative distances (x-axis) from different genomic regions computed using the *bedtools reldist* command. When two genomic tracks are not spatially related, one expects the fraction of relative distance distribution to be uniform. **Figure S12.** Genomic distribution of TFBSs in *A. thaliana*, *C. elegans* and *D. rerio*. Distribution of the proportion of *A. thaliana*, *C. elegans* and *D. rerio* UniBind robust TFBSs overlapping with different types of genomic regions (colors; see legend) across TFs (columns). **Figure S13.** Genomic distribution of TFBSs in *D. melanogaster* and *H. sapiens*. Distribution of the proportion of *D. melanogaster* and *H. sapiens* UniBind robust TFBSs overlapping with different types of genomic regions (colors; see legend) across TFs (columns). **Figure S14**. Genomic distribution of TFBSs in *H. sapiens (continued)* and *M. musculus*. Distribution of the proportion of *H. sapiens* (continued) and *M. musculus* UniBind robust TFBSs overlapping with different types of genomic regions (colors; see legend) across TFs (columns). **Figure S15.** Genomic distribution of TFBSs in *M. musculus (continued)*. Distribution of the proportion of *M. musculus* (continued) UniBind robust TFBSs overlapping with different types of genomic regions (colors; see legend) across TFs (columns). **Figure S16.** Genomic distribution of TFBSs in *R. norvegicus* and *S. cerevisiae*. Distribution of the proportion of *R. norvegicus* and *S. cerevisiae* UniBind robust TFBSs overlapping with different types of genomic regions (colors; see legend) across TFs (columns). **Figure S17.** Enrichment analysis for *H. sapiens* TFBSs in ENCODE cCREs. Barplots representing the expected (grey bars) versus observed (blue bars) overlap lengths (**A**) or number of intersections (**B**) between *H. sapiens* TFBSs from the robust collection and ENCODE cCREs (x-axis). The plots and computed p-values (green: enrichment; orange: depletion) were obtained using the OLOGRAM command of the GTF toolkit. **Figure S18.** Enrichment analysis for *M. musculus* TFBSs in ENCODE cCREs. Barplots representing the expected (grey bars) versus observed (blue bars) overlap lengths (**A**) or number of intersections (**B**) between *M. musculus* TFBSs from the robust collection and ENCODE cCREs (x-axis). The plots and computed p-values (green: enrichment; orange: depletion) were obtained using the OLOGRAM command of the GTF toolkit. **Figure S19.** Enrichment analysis for *H. sapiens* CRMs in ENCODE cCREs. Barplots representing the expected (grey bars) versus observed (blue bars) overlap lengths (**A**) or number of intersections (**B**) between *H. sapiens* CRMs from the robust collection and ENCODE cCREs (x-axis). The plots and computed p-values (green: enrichment; orange: depletion) were obtained using the OLOGRAM command of the GTF toolkit. **Figure S20.** Enrichment analysis for *M. musculus* CRMs in ENCODE cCREs. Barplots representing the expected (grey bars) versus observed (blue bars) overlap lengths (**A**) or number of intersections (**B**) between *M. musculus* CRMs from the robust collection and ENCODE cCREs (x-axis). The plots and computed p-values (green: enrichment; orange: depletion) were obtained using the OLOGRAM command of the GTF toolkit. **Figure S21.** Relative distance distributions between CRMs and ENCODE cCREs. Fraction of CRMs in the UniBind robust collection (y-axis) with respect to increasing relative distances (x-axis) from ENCODE cCREs computed using the *bedtools reldist* command for human (**A**) and mouse (**B**). When two genomic tracks are not spatially related, one expects the fraction of relative distance distribution to be uniform. **Figure S22.** Correlation between enhancer activity and TF binding. For each enhancer predicted using Cap Analysis of Gene Expression (CAGE) by the FANTOM5 consortium, we computed the number of TFs with overlapping TFBSs in the robust collection of UniBind (x-axis). The figure provides, for each value of the number of TFs found to bind in enhancers, the median (blue line) together with the 10th to 90th percentiles (grey area) of tissue specific activity of these enhancers. The expression measures were derived from CAGE (capturing enhancer RNA expression). The specificity of activity (y-axis) is provided within the [0; 1] range with 0 representing ubiquitous enhancer activity and 1 exclusive expression activity.

## Data Availability

All the results are freely available on the UniBind website at https://unibind.uio.no. Pointers to the input data used from GTRD [5] and ReMap [26] are provided on the UniBind website. Genome browser tracks can also be accessed through the public hub search at the UCSC genome browser and at https://trackhubregistry.org. The code for the ChIP-eat pipeline and the enrichment tool is publicly accessible at https://bitbucket.org/CBGR/chip-eat/ and https://bitbucket.org/CBGR/unibind_enrichment/, respectively. The LOLA databases for the enrichment tool are available to the public at 10.5281/zenodo.4704641. Finally, the source code of the UniBind web-application together with installation instructions are available at https://bitbucket.org/CBGR/unibind.

## Abbreviations

API: Application programming interface
ATAC-seq: Assay for transposase-accessible chromatin using sequencing
bp: Base pairs
CAGE: Cap analysis of gene expression
cCRE: Candidate cis-regulatory element
ChExMix: ChIP-exo mixture model
ChIP: Chromatin immunoprecipitation
ChIP-seq: Chromatin immunoprecipitation followed by sequencing
CREAM: Clustering of genomic regions analysis method
CRM: Cis-regulatory module
DAMO: Discriminative additive model optimization
DBD: DNA binding domain
dELS: Distal enhancer-like signature
DNA: Deoxyribonucleic acid
DNase-seq: DNase I hypersensitive sites sequencing
ELS: Enhancer-like signature
ENCODE: Encyclopedia of DNA elements
eRNA: Enhancer ribonucleic acid
ER +: Estrogen receptor positive
GEO: Gene expression omnibus
GTRD: Gene transcription regulation database
LOLA: Locus overlap analysis
MACS: Model-based analysis of ChIP-seq
OLOGRAM: OverLap Of Genomic Regions Analysis using Monte Carlo
PFM: Position frequency matrix
pELS: Proximal enhancer-like signature
PLS: Promoter-like signature
PWM: Position weight matrix
QC: Quality control
REST: Representational state transfer
RNA: Ribonucleic acid
SCREEN: Search candidate regulatory elements by ENCODE
TF: Transcription factor
TFBS: Transcription factor binding site
TFFM: Transcription factor flexible model
TSS: Transcription start site
UCSC: University of California, Santa Cruz
