# Supplementary material for: UniBind: maps of high-confidence direct TF-DNA interactions across nine species
Source: BMC Genomics. 2021 Jun 26;22:482. doi: 10.1186/s12864-021-07760-6 (PMC8236138; doi:10.1186/s12864-021-07760-6)
Supplement: Supplementary file 1 — Additional file 1: Table S1. Overview of the permissive collection. Table providing the number of datasets, TFs, cell / tissue types, and TFBSs in the permissive collection of UniBind. The number of TFBSs was computed as the number of unique instances of genomic loci bound by a TF. Table S2. Overview of the robust collection. Table providing the number of datasets, TFs, cell /tissue types, and TFBSs in the robust collection of UniBind. The number of TFBSs was computed as the number of unique instances of genomic loci bound by a TF. Figure S1. Visual overview of the permissive collection. (A) Barplots showing the number of TFs (dark orange), TFBSs (green), datasets (blue), and cell and tissue types (light orange) stored in the permissive collection of UniBind for each analyzed species. All values are log10-transformed. (B) Distribution of the percentages of the genomes covered by robust TFBSs in each species (one color per species, see legend). Figure S2. Relationship between number of datasets and genome coverage. Scatter plots representing the percentage of genome coverage (y-axes) with respect to the number of datasets in the permissive (A) and robust (C) collections or the number of TFs in the permissive (B) and robust (D) collection (x-axes). Each colored point in each panel represents the data associated to one species (see legend for color coding). Figure S3. The UniBind 2021 compressed and robust tracks with all TFBSs from the robust human collection. An example of a random genomic locus showing the comparison between the original and archetypal TFBSs. The tracks shown are, from top to bottom: RefSeq track with the first intron of the human TTC6 gene, the UniBind compressed track with archetypal TFBSs, and the UniBind robust track showing all TFBSs at the same location. Figure S4. Evolutionary conservation at human and mouse robust CRMs. Distributions of the average base-pair evolutionary conservation scores (phyloP and phastCons scores using multi-species ge [file 12864_2021_7760_MOESM1_ESM.pdf]

Supplementary Tables

Organism	Number of datasets	Number of TFs	Number of cells / tissue types	Number of TFBSs	Number of CRMs
A. thaliana	121	41	27	181,509	2,226
C. elegans	182	31	17	116,018	604
D. rerio	12	8	8	90,455	1,136
D. melanogaster	264	77	42	181,359	1,623
H. sapiens	4,659	324	570	37,834,304	114,059
M. musculus	4,248	319	628	33,174,655	71,061
R. norvegicus	66	15	15	1,055,551	7,018
S. cerevisiae	99	25	9	7,687	166
S. pombe	3	1	1	46	0
**Total**	9,654	841	1,316	72,641,584	197,893

Supplementary Table 1. **Overview of the permissive collection**. Table providing the number of datasets, TFs, cell / tissue types, and TFBSs in the permissive collection of UniBind. The number of TFBSs was computed as the number of unique instances of genomic loci bound by a TF.

Organism	Number of datasets	Number of TFs	Number of cells / tissue types	Number of TFBSs	Number of CRMs
A. thaliana	78	33	22	169,649	1,262
C. elegans	91	21	12	93,138	503
D. rerio	6	5	4	44,187	617
D. melanogaster	109	22	28	95,715	1,162
H. sapiens	3,478	268	501	29,276,761	104,143
M. musculus	3,070	269	512	25,263,323	73,919
R. norvegicus	41	12	13	924,254	16,163
S. cerevisiae	29	14	4	3,691	121
**Total**	6,902	644	1,096	55,870,115	197,890

Supplementary Table 2. **Overview of the robust collection**. Table providing the number of datasets, TFs, cell / tissue types, and TFBSs in the robust collection of UniBind.The number of TFBSs was computed as the number of unique instances of genomic loci bound by a TF.

Supplementary Figures

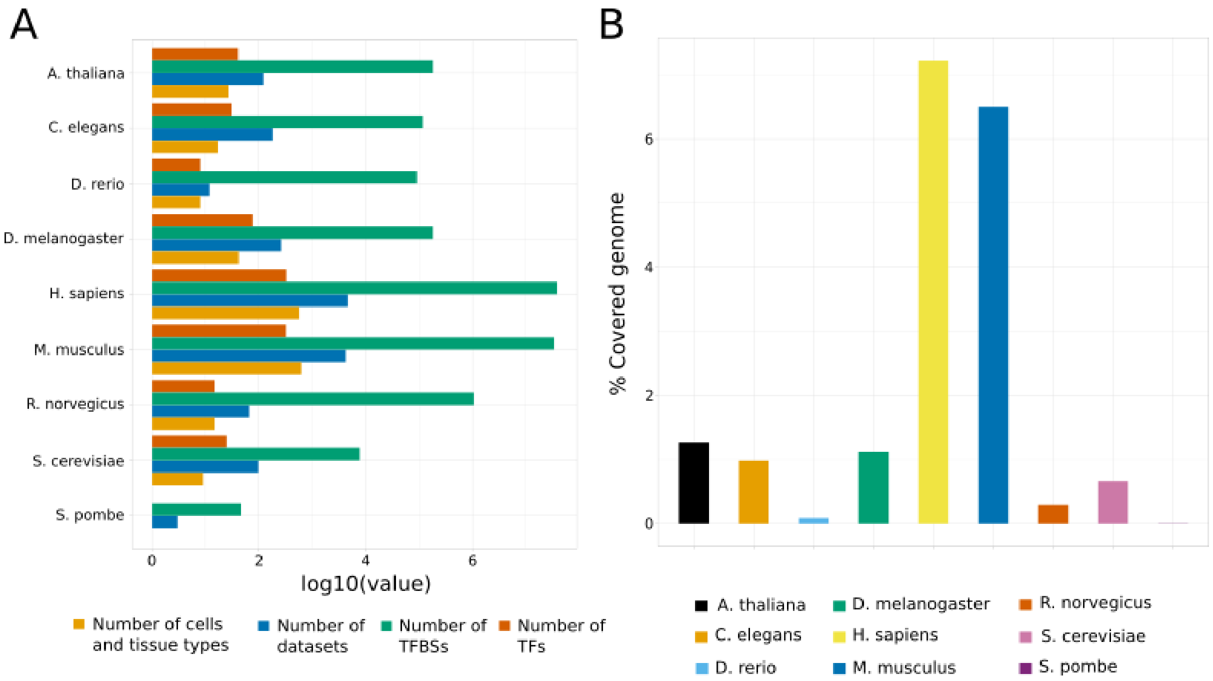

Supplementary Figure 1. **Visual overview of the permissive collection**. **Figure 1**. (**A**) Barplots showing the number of TFs (dark orange), TFBSs (green), datasets (blue), and cell and tissue types (light orange) stored in the permissive collection of UniBind for each analyzed species. All values are log10-transformed. (**B**) Distribution of the percentages of the genomes covered by robust TFBSs in each species (one color per species, see legend).

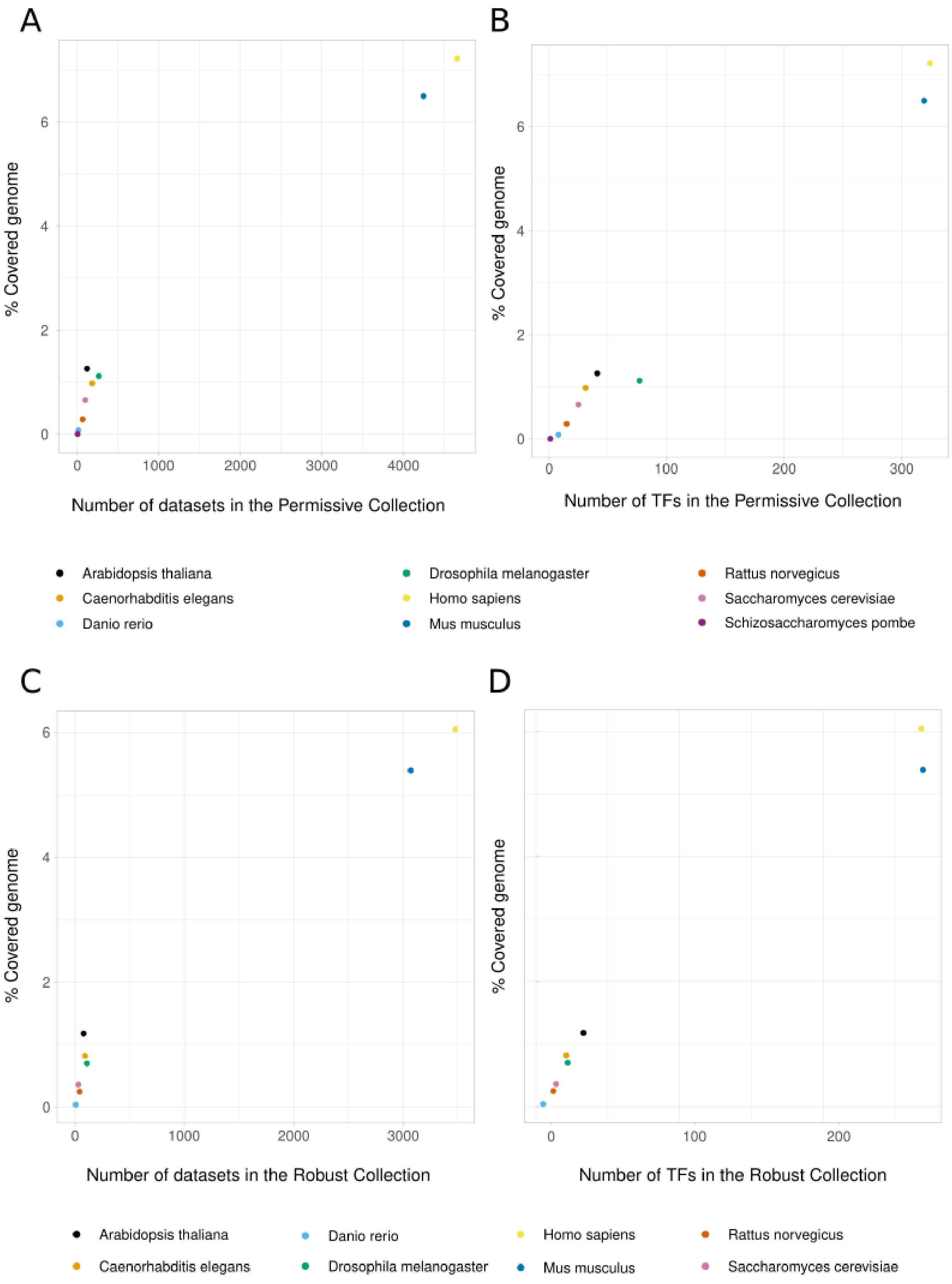

Supplementary Figure 2. **Relationship between number of datasets and genome coverage**. Scatter plots representing the percentage of genome coverage (y-axes) with respect to the number of datasets in the permissive (**A**) and robust (**C**) collections or the number of TFs in the permissive (**B**) and robust (**D**) collection (x-axes). Each colored point in each panel represents the data associated to one species (see legend for color coding).

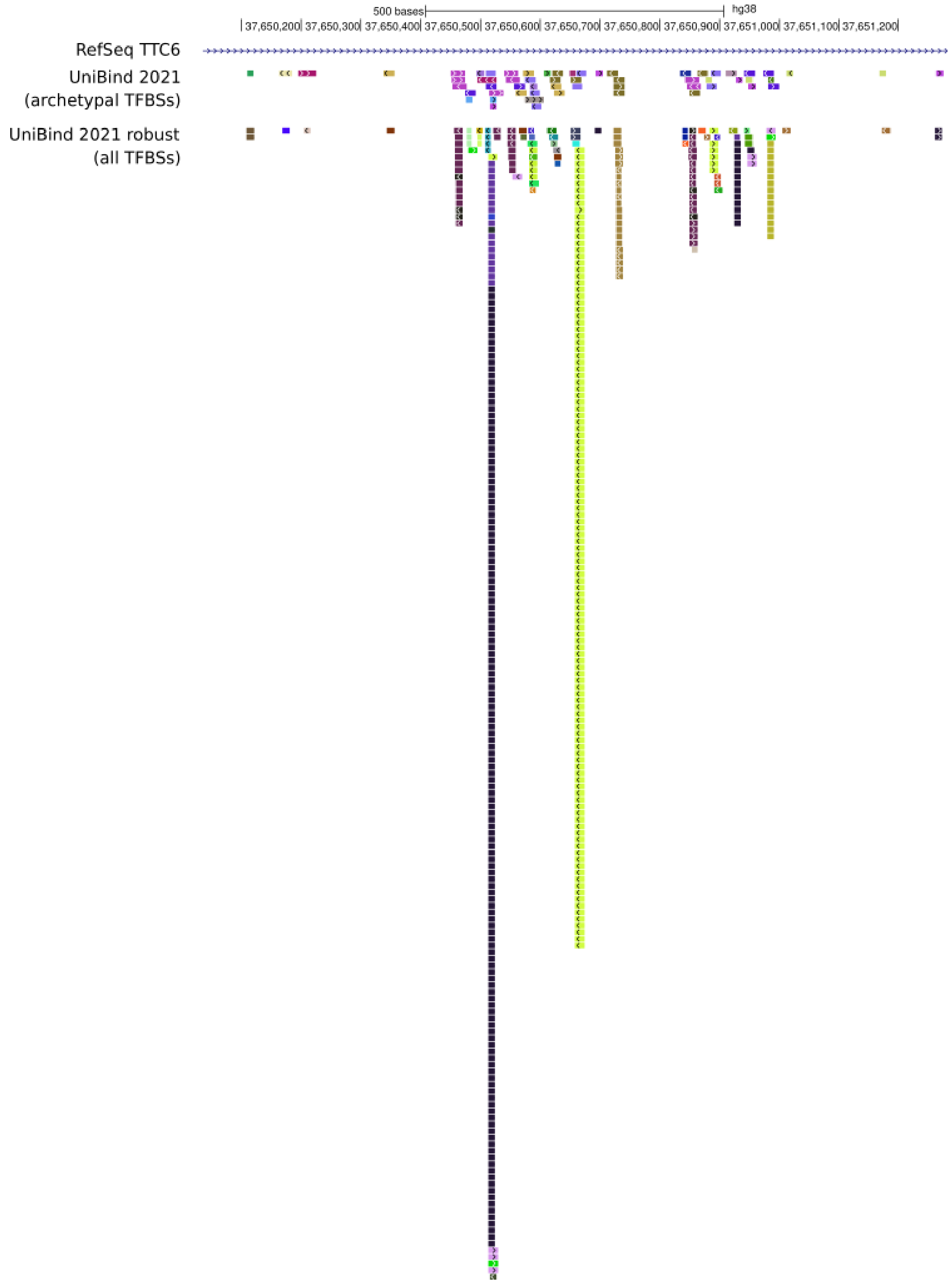

Supplementary Figure 3. **The UniBind 2021 compressed and robust tracks with all TFBSs from the robust human collection**. An example of a random genomic locus showing the comparison between the original and archetypal TFBSs. The tracks shown are, from top to bottom: RefSeq track with the first intron of the human TTC6 gene, the UniBind compressed track with archetypal TFBSs, and the UniBind robust track showing all TFBSs at the same location.

A

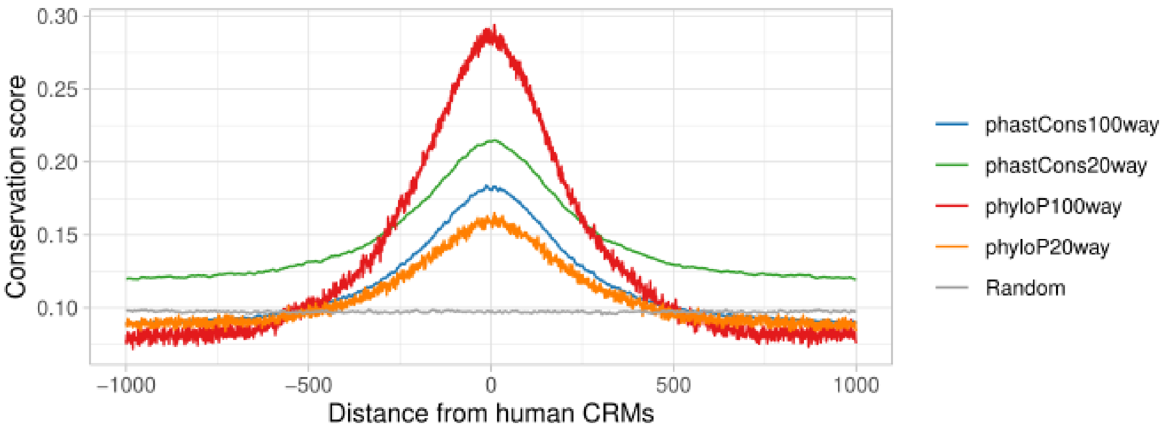

B

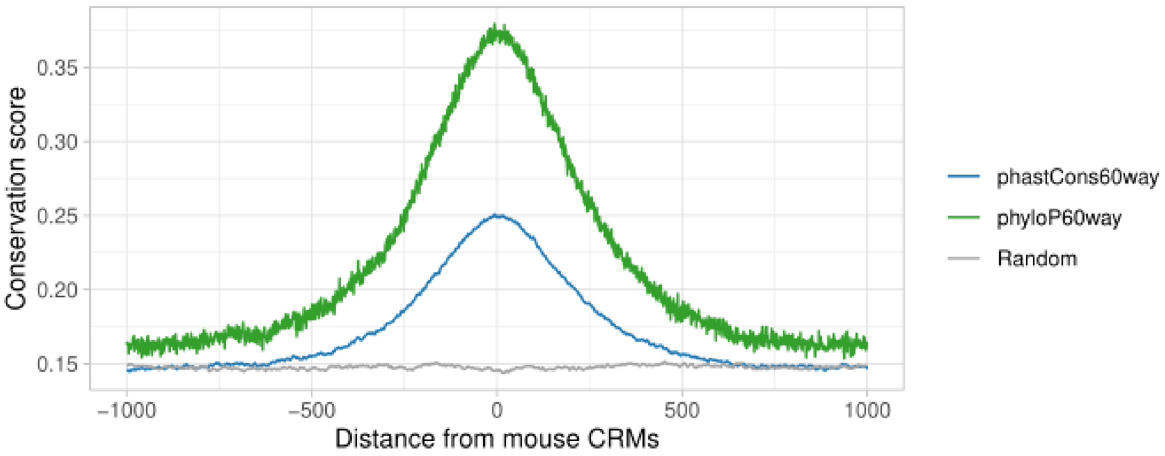

Supplementary Figure 4. **Evolutionary conservation at human and mouse robust CRMs**. Distributions of the average base-pair evolutionary conservation scores (phyloP and phastCons scores using multi-species genome alignments, see legend) at regions centered around UniBind human (**A**) and mouse (**B**) CRMs from the robust collection. Conservation of random CRMs was obtained by shuffling the original CRMs and obtaining the conservation score of the new regions.

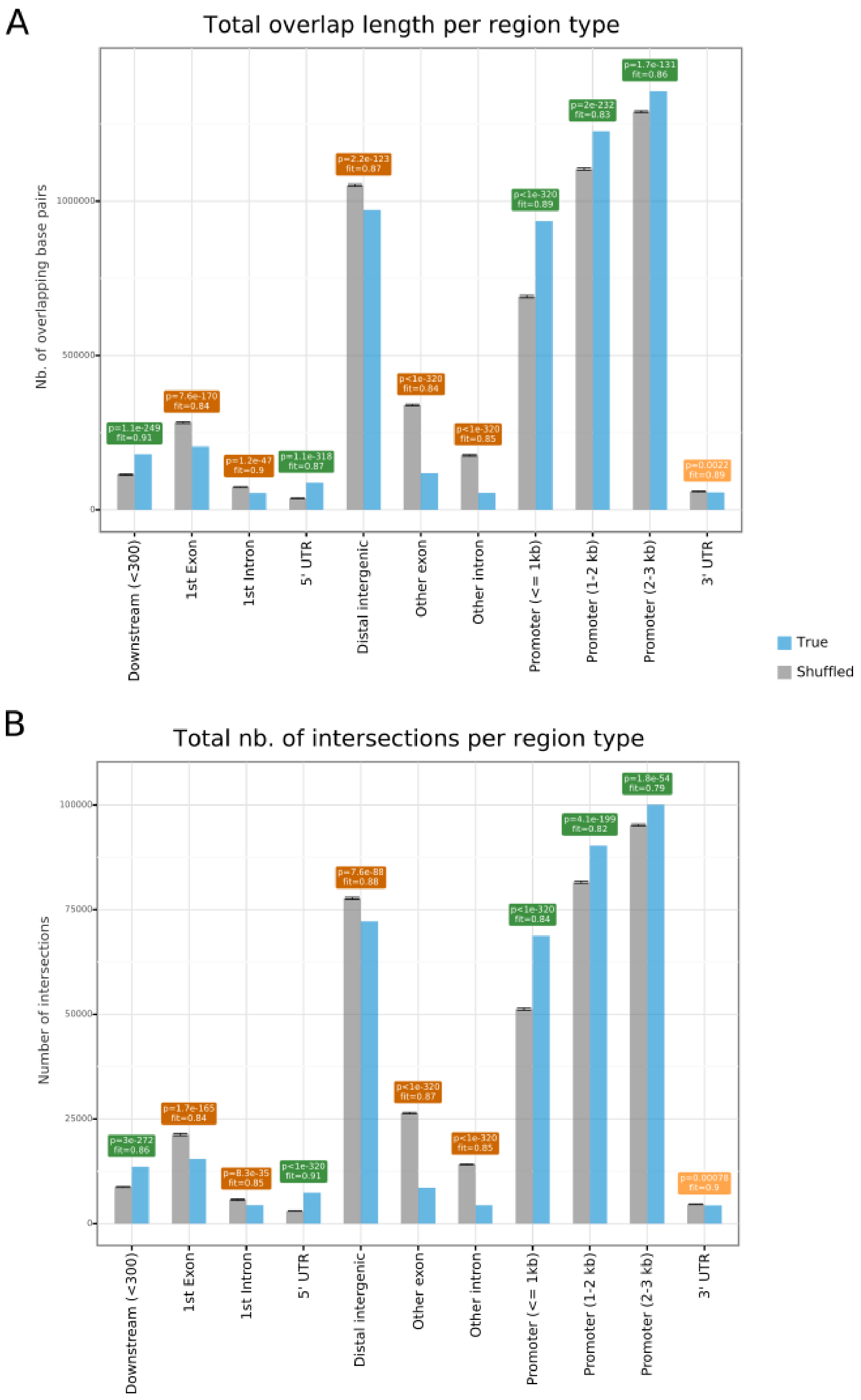

Supplementary Figure 5. **Enrichment analysis for *A. thaliana* TFBSs in genomic regions**. Barplots representing the expected (grey bars) versus observed (blue bars) overlap lengths (**A**) or number of intersections (**B**) between *A. thaliana* TFBSs from the robust collection and genomic annotations (x-axis). The plots and computed p-values (green: enrichment; orange: depletion) were obtained using the OLOGRAM command of the GTF toolkit.

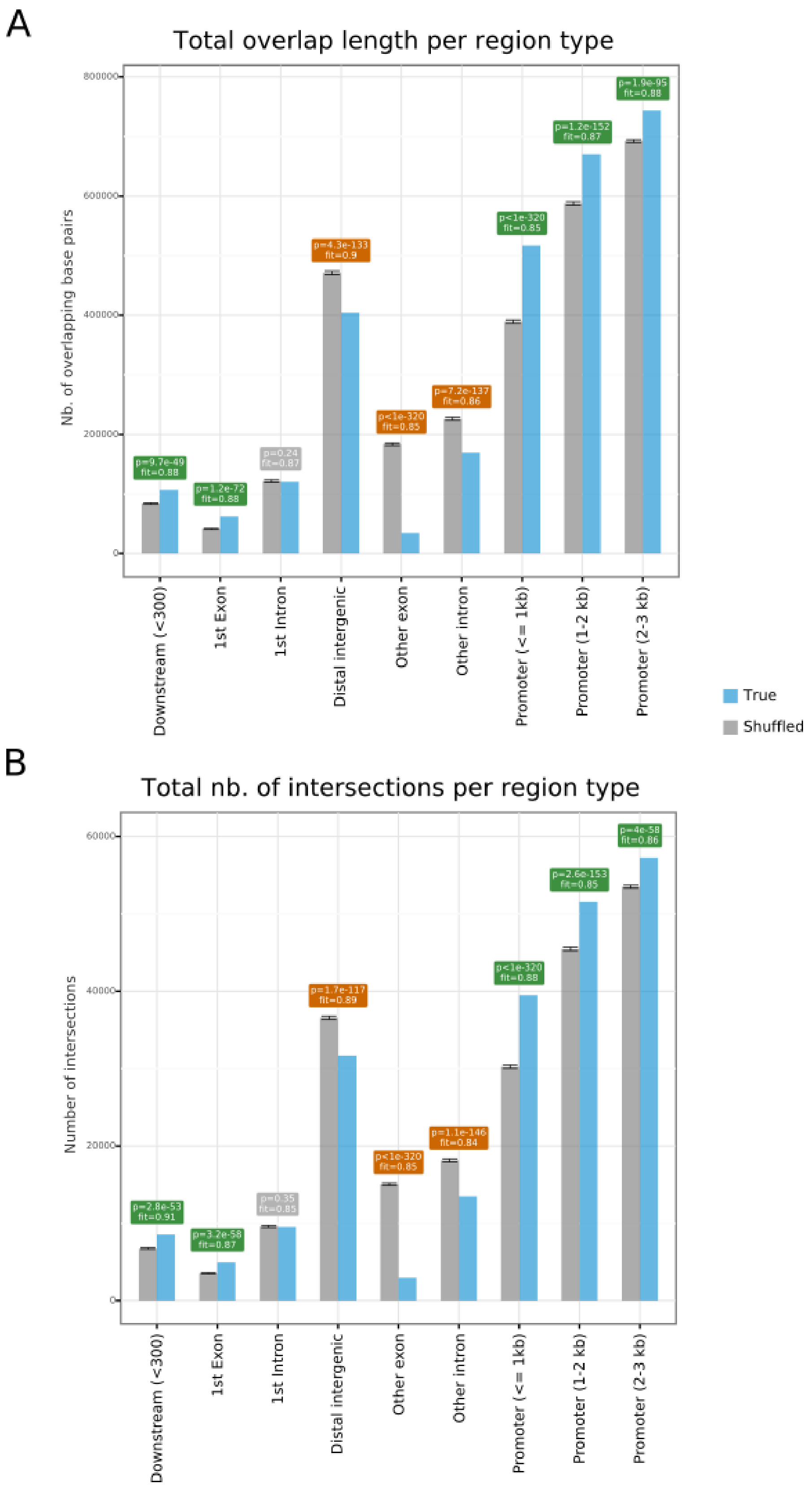

Supplementary Figure 6. **Enrichment analysis for *C. elegans* TFBSs in genomic regions**. Barplots representing the expected (grey bars) versus observed (blue bars) overlap lengths (**A**) or number of intersections (**B**) between *C. elegans* TFBSs from the robust collection and genomic annotations (x-axis). The plots and computed p-values (green: enrichment; orange: depletion) were obtained using the OLOGRAM command of the GTF toolkit.

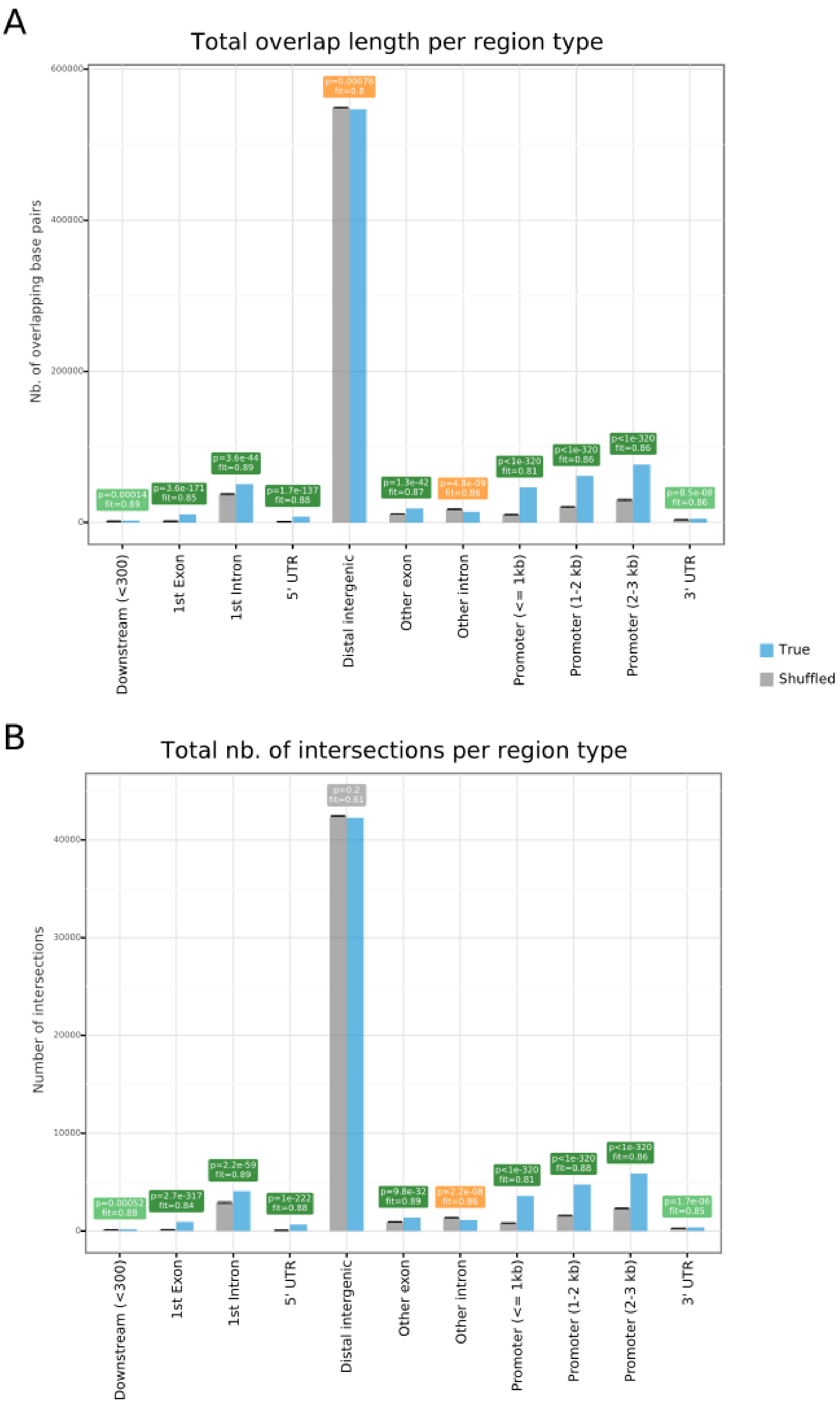

Supplementary Figure 7. **Enrichment analysis for *D. rerio* TFBSs in genomic regions**. Barplots representing the expected (grey bars) versus observed (blue bars) overlap lengths (**A**) or number of intersections (**B**) between *D. rerio* TFBSs from the robust collection and genomic annotations (x-axis). The plots and computed p-values (green: enrichment; orange: depletion) were obtained using the OLOGRAM command of the GTF toolkit.

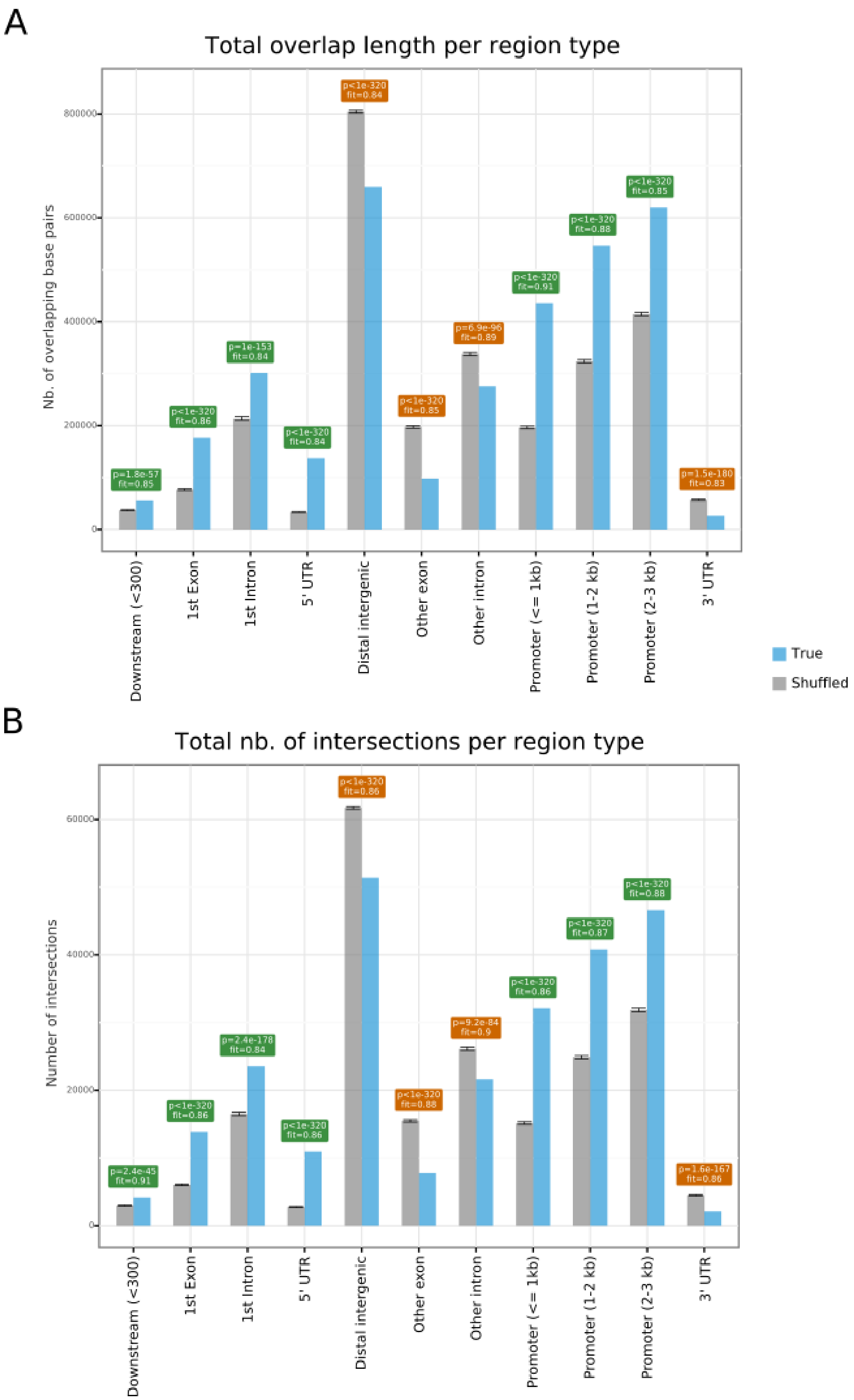

Supplementary Figure 8. Enrichment analysis for *D. melanogaster* TFBSs in genomic regions. Barplots representing the expected (grey bars) versus observed (blue bars) overlap lengths (**A**) or number of intersections (**B**) between *D. melanogaster* TFBSs from the robust collection and genomic annotations (x-axis). The plots and computed p-values (green: enrichment; orange: depletion) were obtained using the OLOGRAM command of the GTF toolkit.

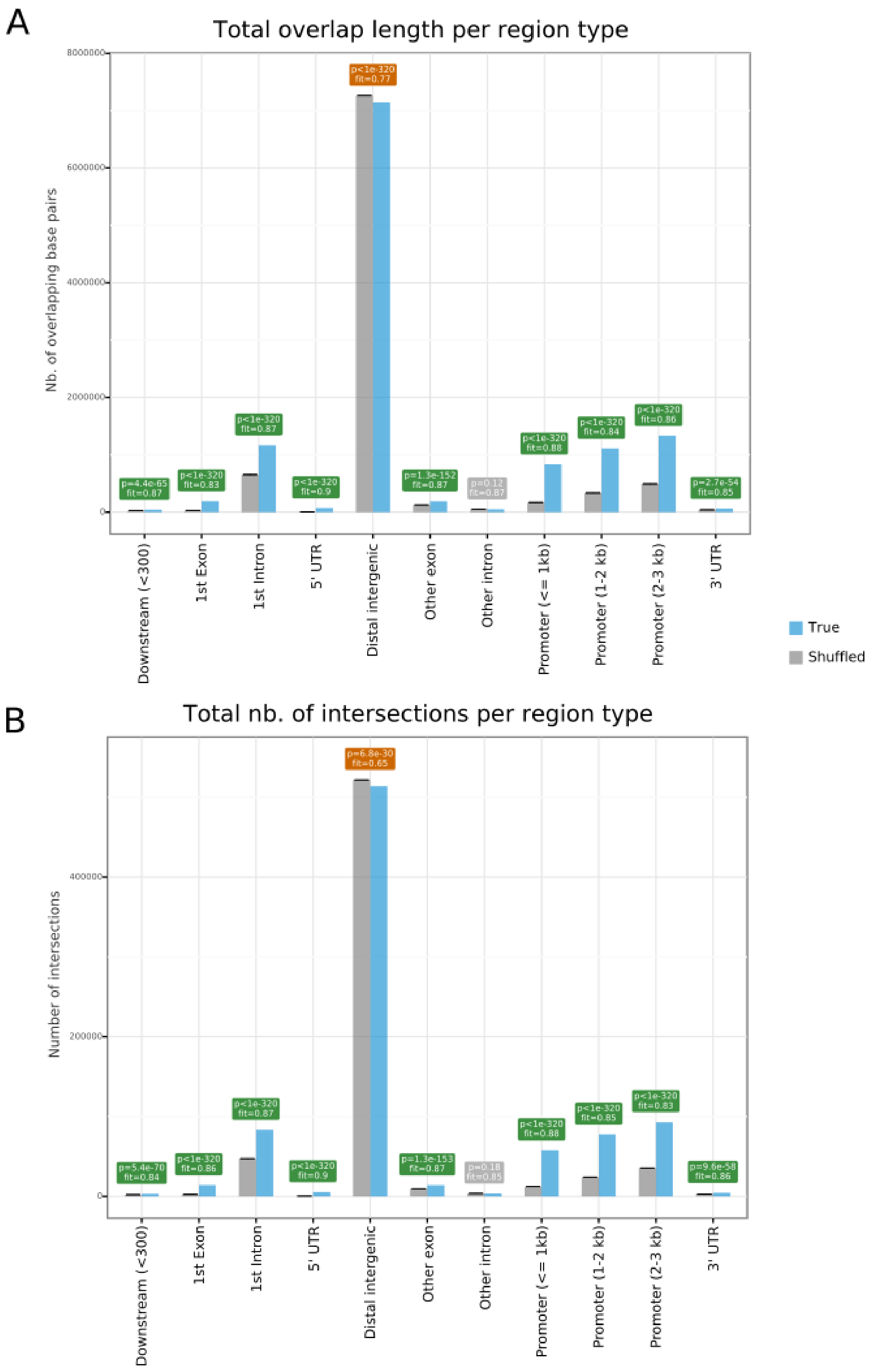

Supplementary Figure 9. **Enrichment analysis for *R. norvegicus* TFBSs in genomic regions**. Barplots representing the expected (grey bars) versus observed (blue bars) overlap lengths (**A**) or number of intersections (**B**) between *R. norvegicus* TFBSs from the robust collection and genomic annotations (x-axis). The plots and computed p-values (green: enrichment; orange: depletion) were obtained using the OLOGRAM command of the GTF toolkit.

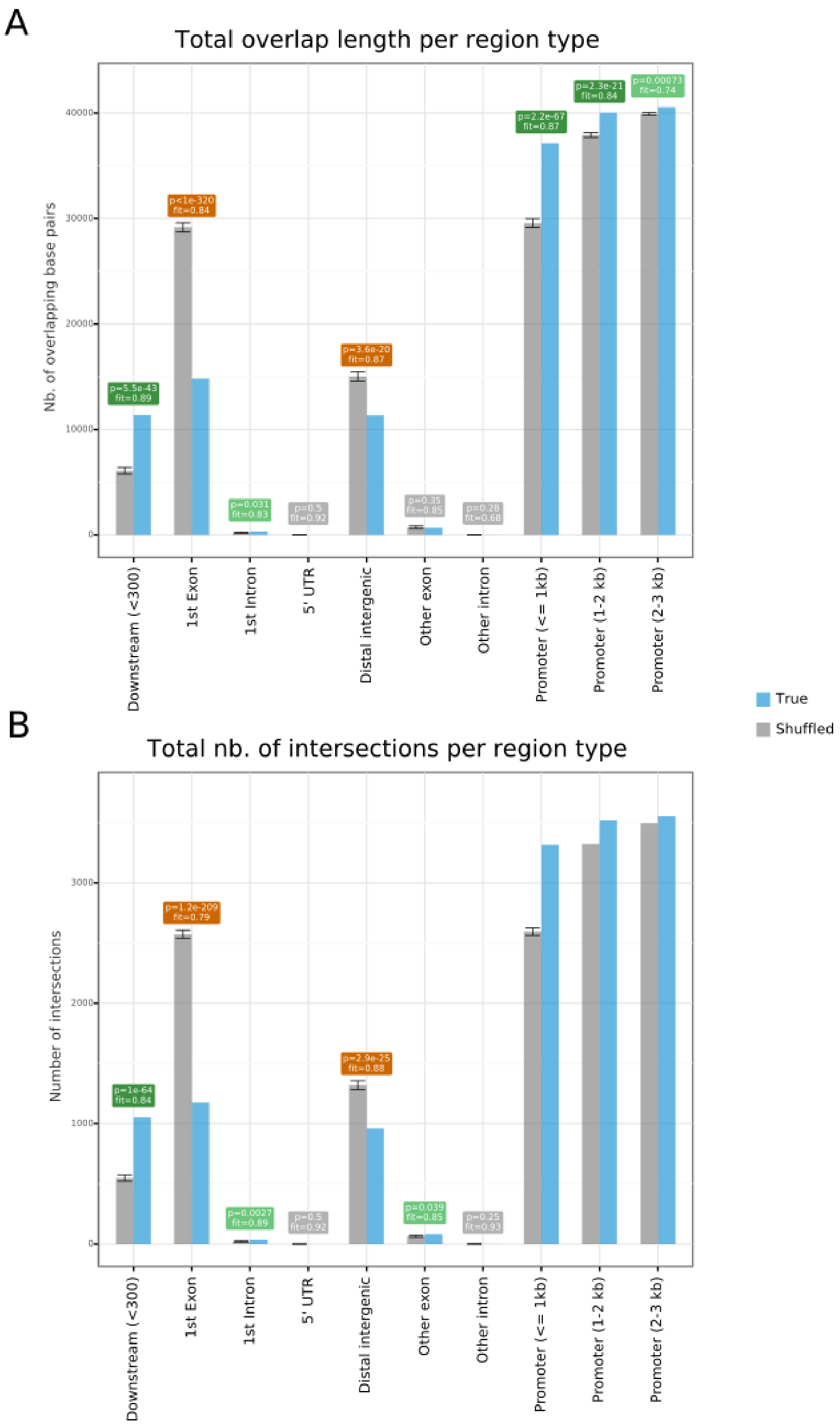

Supplementary Figure 10. **Enrichment analysis for *S. cerevisae* TFBSs in genomic regions**. Barplots representing the expected (grey bars) versus observed (blue bars) overlap lengths (**A**) or number of intersections (**B**) between *S. cerevisae* TFBSs from the robust collection and genomic annotations (x-axis). The plots and computed p-values (green: enrichment; orange: depletion) were obtained using the OLOGRAM command of the GTF toolkit.

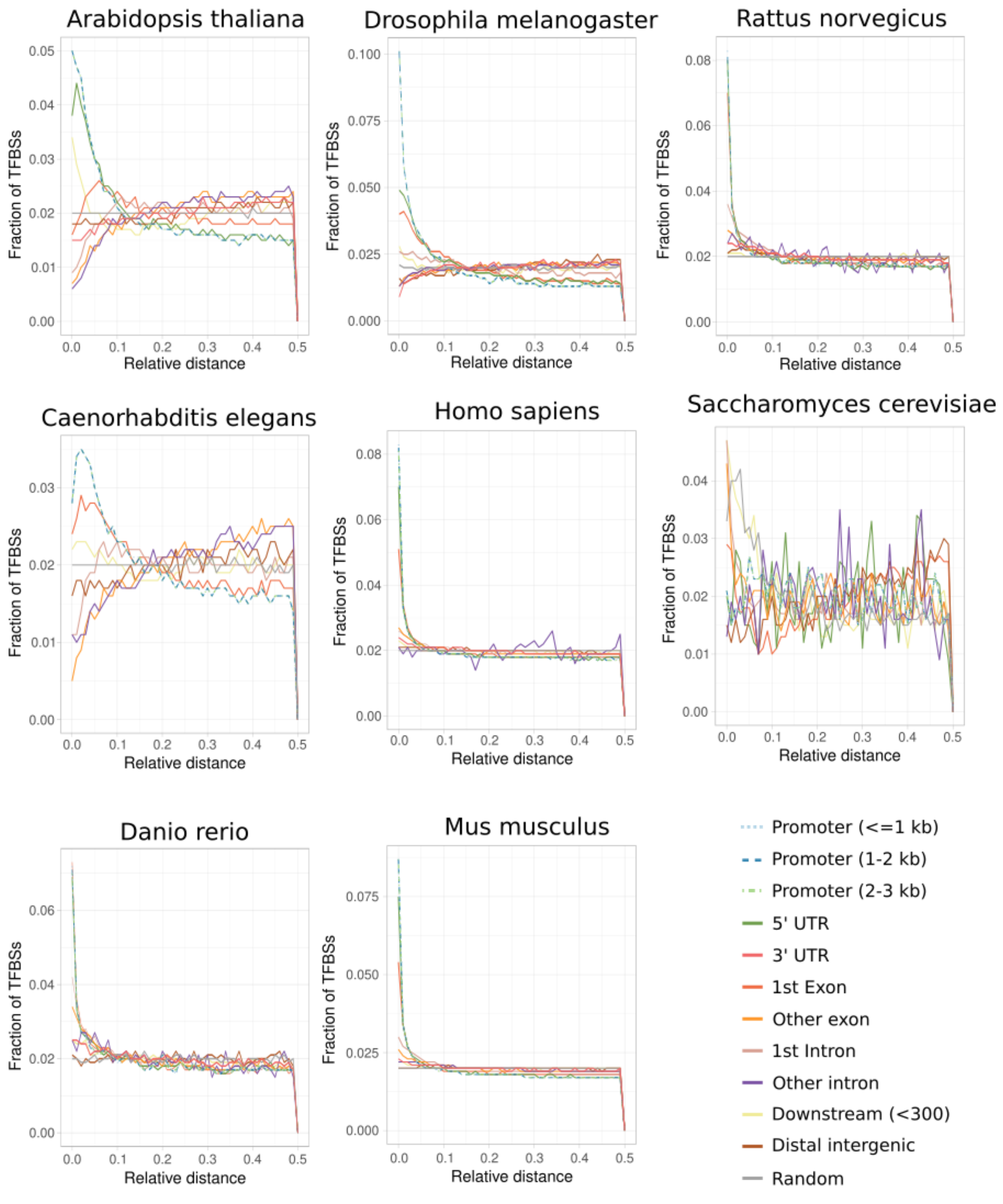

Supplementary Figure 11. **Analysis of the overlap of robust TFBSs with respect to genomic annotations in all species in UniBind**. Fraction of TFBSs in the UniBind robust collection (y-axis) with respect to increasing relative distances (x-axis) from different genomic regions computed using the *bedtools reldist* command. When two genomic tracks are not spatially related, one expects the fraction of relative distance distribution to be uniform.

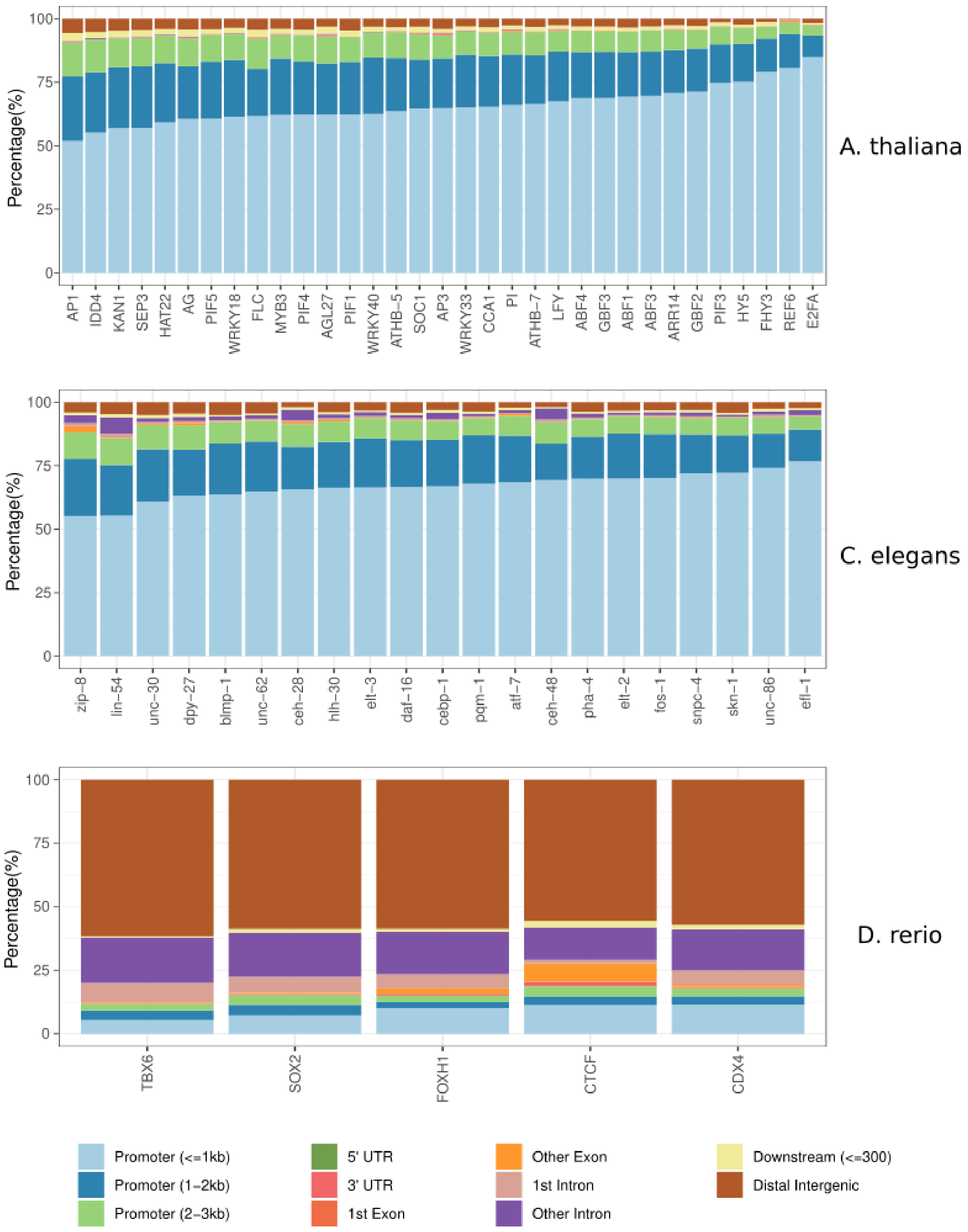

Supplementary Figure 12. Genomic distribution of TFBSs in *A. thaliana*, *C. elegans* and *D. rerio*. Distribution of the proportion of *A. thaliana*, *C. elegans* and *D. rerio* UniBind robust TFBSs overlapping with different types of genomic regions (colors; see legend) across TFs (columns).

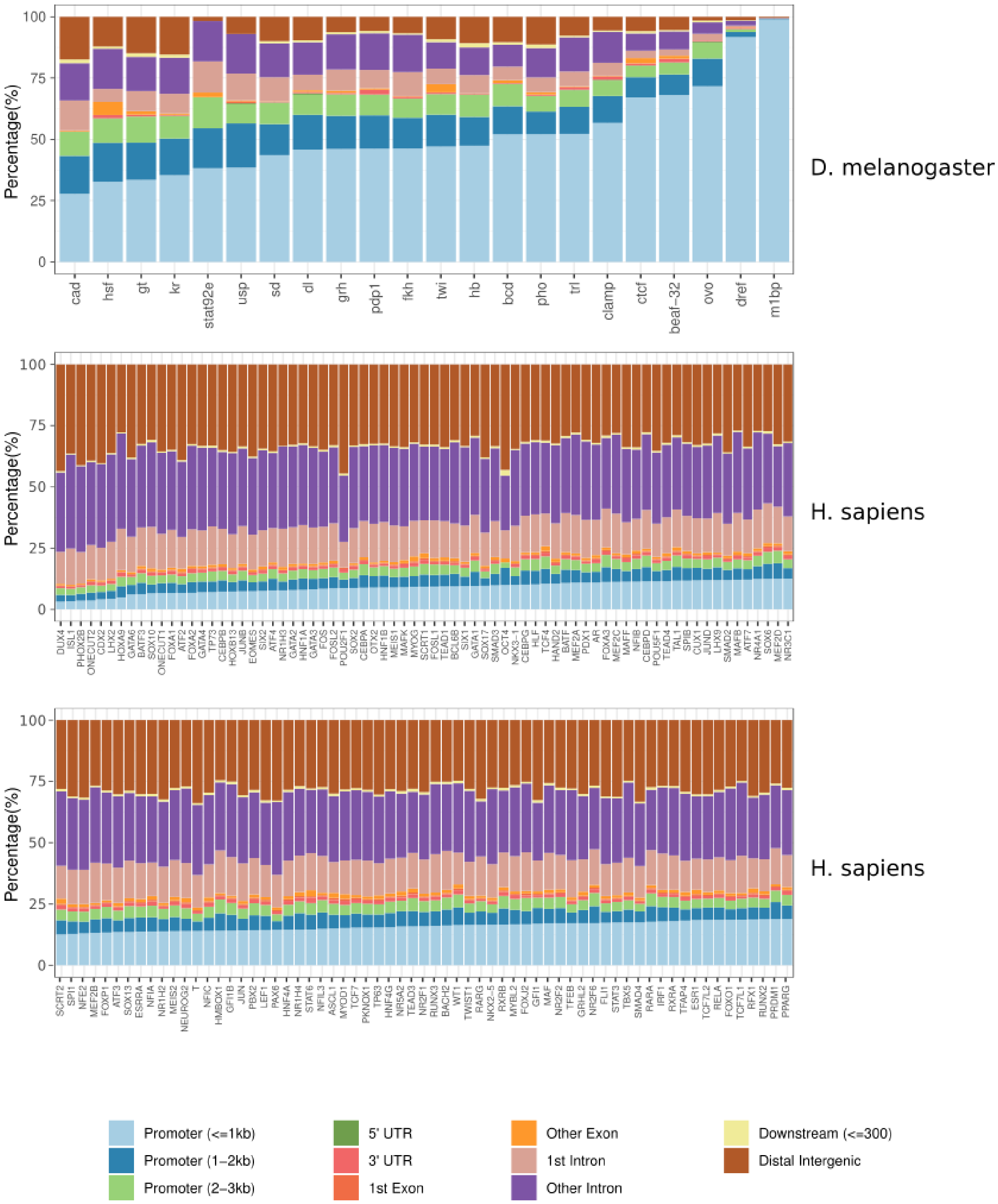

Supplementary Figure 13. Genomic distribution of TFBSs in *D. melanogaster* and *H. sapiens*. Distribution of the proportion of *D. melanogaster* and *H. sapiens* UniBind robust TFBSs overlapping with different types of genomic regions (colors; see legend) across TFs (columns).

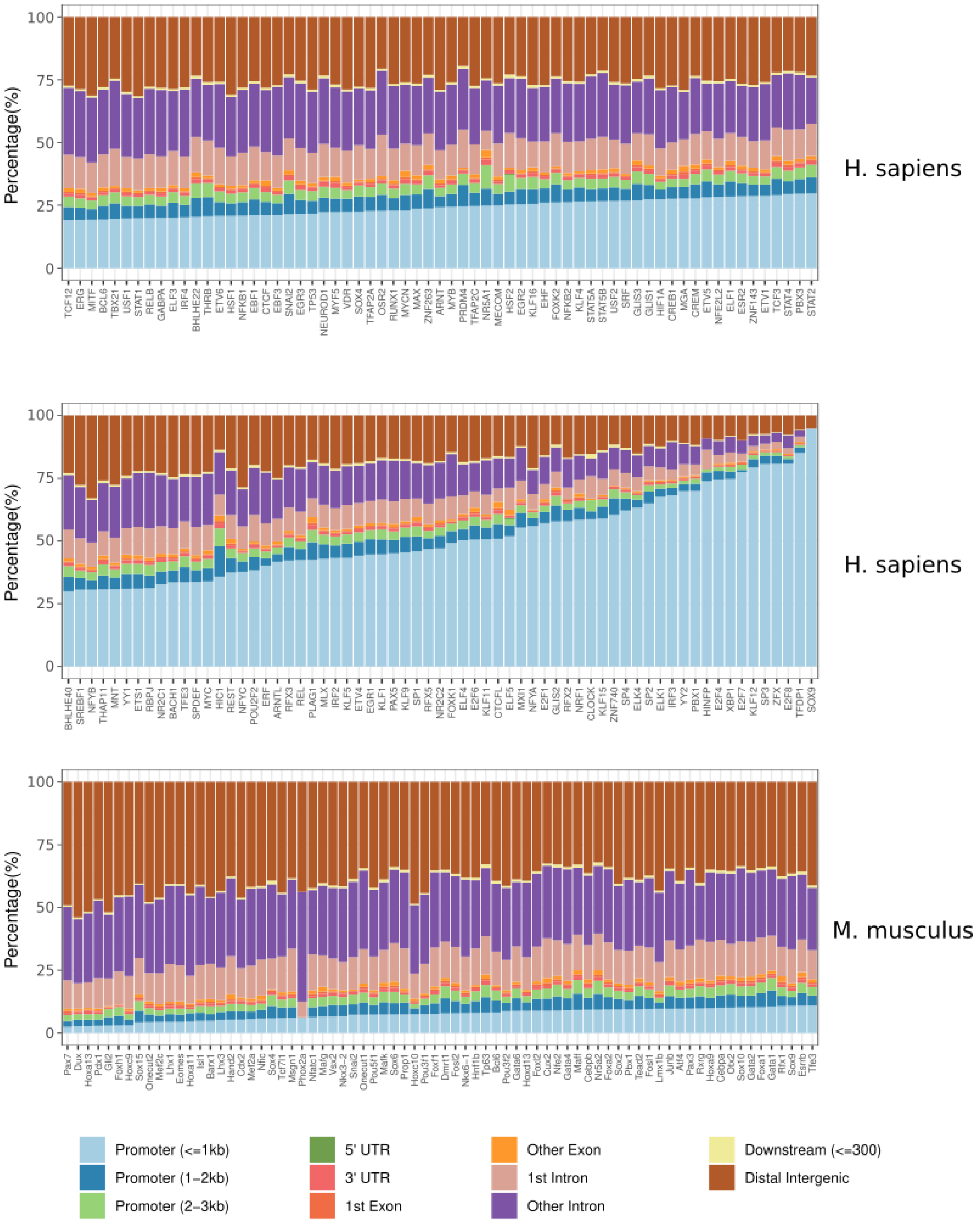

Supplementary Figure 14. Genomic distribution of TFBSs in *H. sapiens (continued)* and *M. musculus*. Distribution of the proportion of *H. sapiens* (continued) and *M. musculus* UniBind robust TFBSs overlapping with different types of genomic regions (colors; see legend) across TFs (columns).

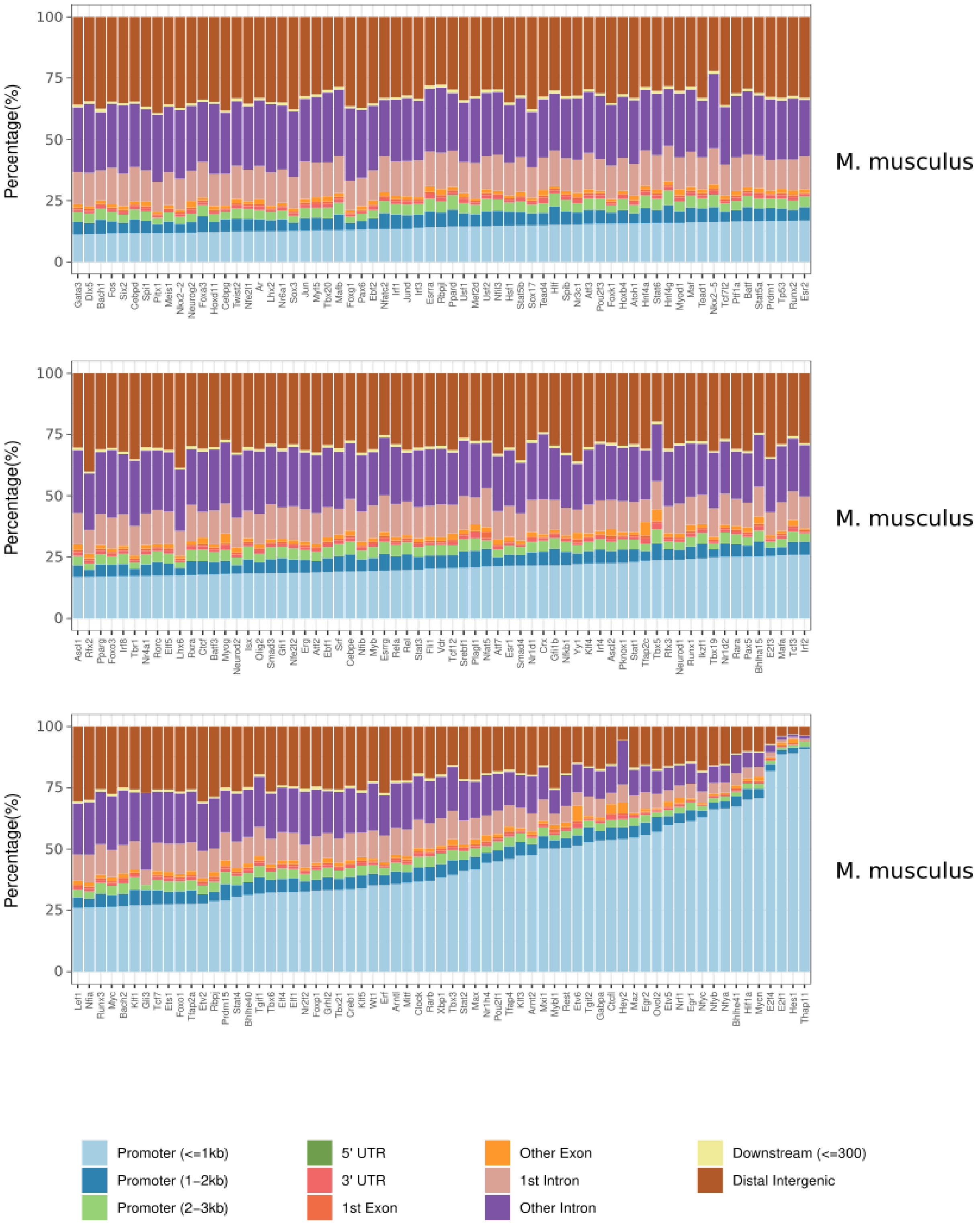

Supplementary Figure 15. Genomic distribution of TFBSs in *M. musculus (continued)*. Distribution of the proportion of *M. musculus* (continued) UniBind robust TFBSs overlapping with different types of genomic regions (colors; see legend) across TFs (columns).

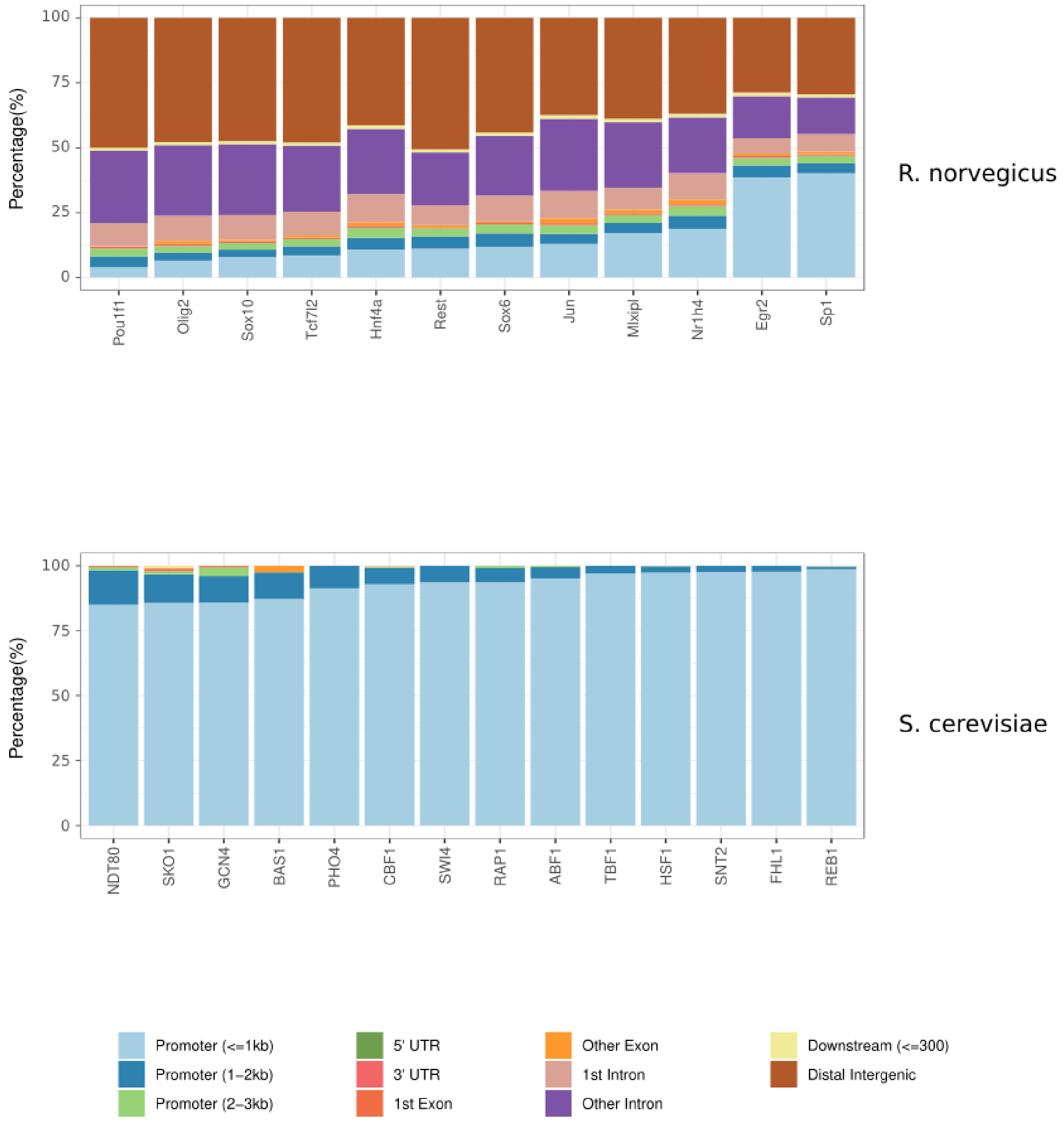

Supplementary Figure 16. Genomic distribution of TFBSs in *R. norvegicus* and *S. cerevisiae*. Distribution of the proportion of *R. norvegicus* and *S. cerevisiae* UniBind robust TFBSs overlapping with different types of genomic regions (colors; see legend) across TFs (columns).

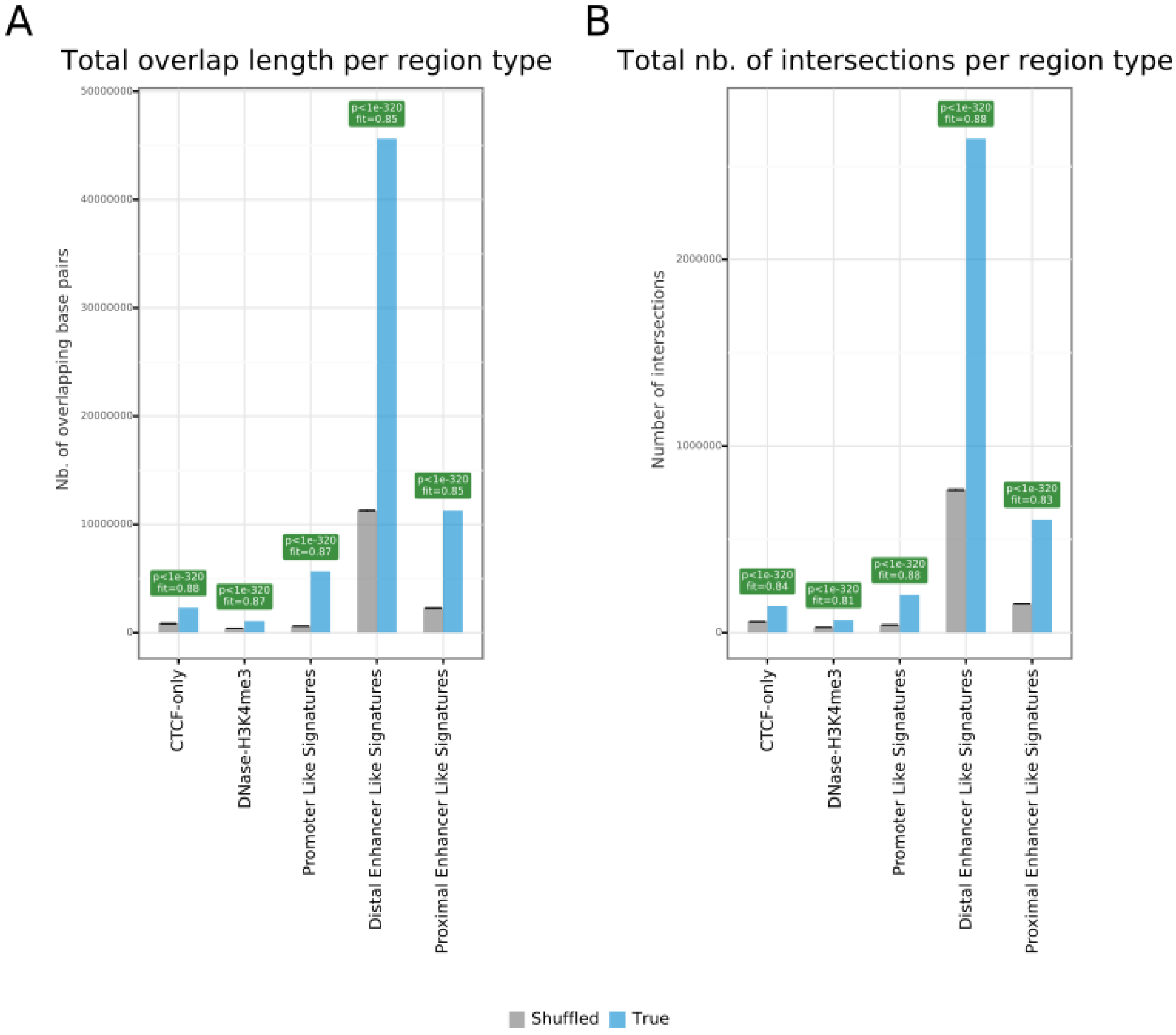

Supplementary Figure 17. **Enrichment analysis for *H. sapiens* TFBSs in ENCODE cCREs**. Barplots representing the expected (grey bars) versus observed (blue bars) overlap lengths (**A**) or number of intersections (**B**) between *H. sapiens* TFBSs from the robust collection and ENCODE cCREs (x-axis). The plots and computed p-values (green: enrichment; orange: depletion) were obtained using the OLOGRAM command of the GTF toolkit.

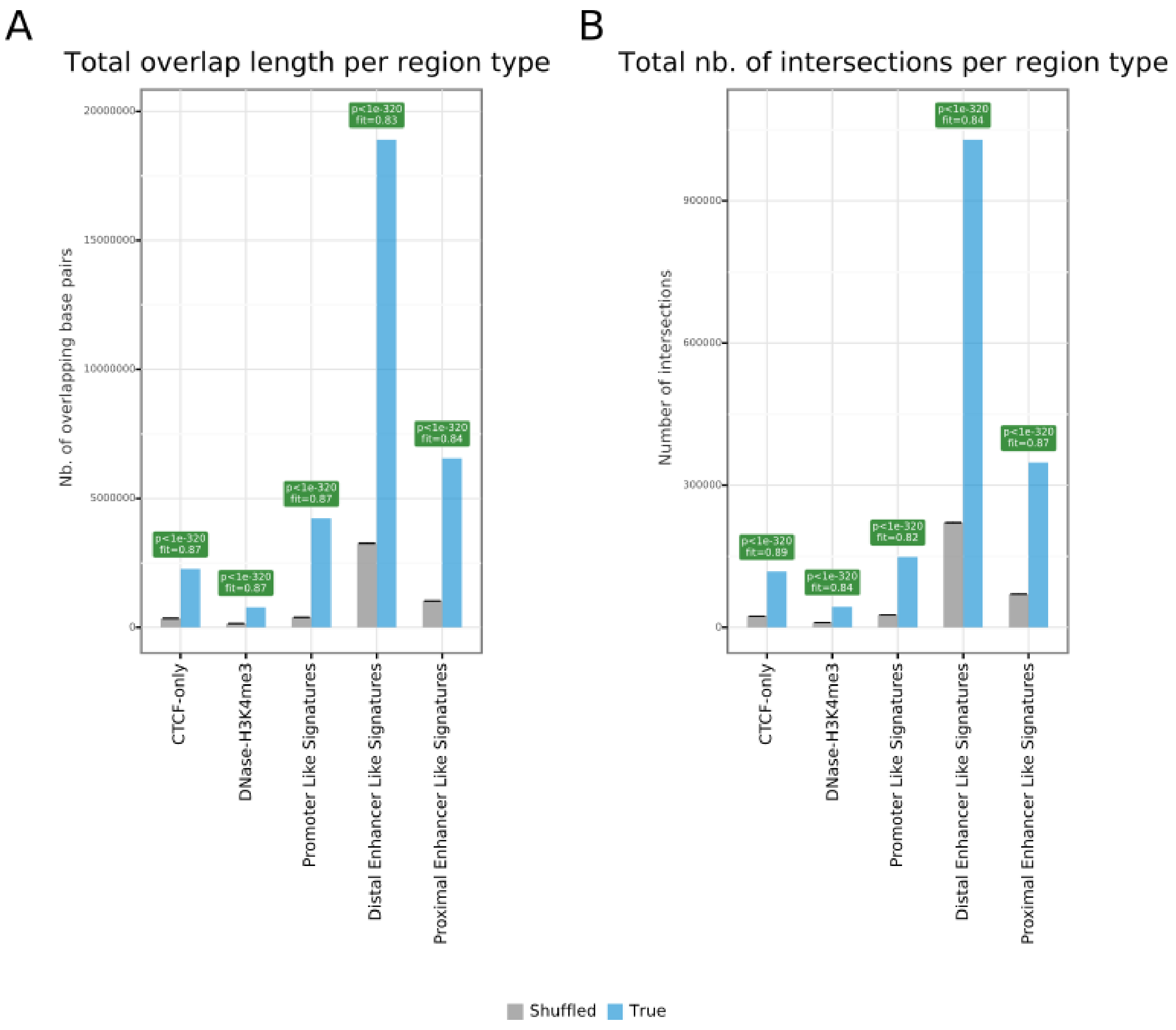

Supplementary Figure 18. **Enrichment analysis for *M. musculus* TFBSs in ENCODE cCREs**. Barplots representing the expected (grey bars) versus observed (blue bars) overlap lengths (**A**) or number of intersections (**B**) between *M. musculus* TFBSs from the robust collection and ENCODE cCREs (x-axis). The plots and computed p-values (green: enrichment; orange: depletion) were obtained using the OLOGRAM command of the GTF toolkit.

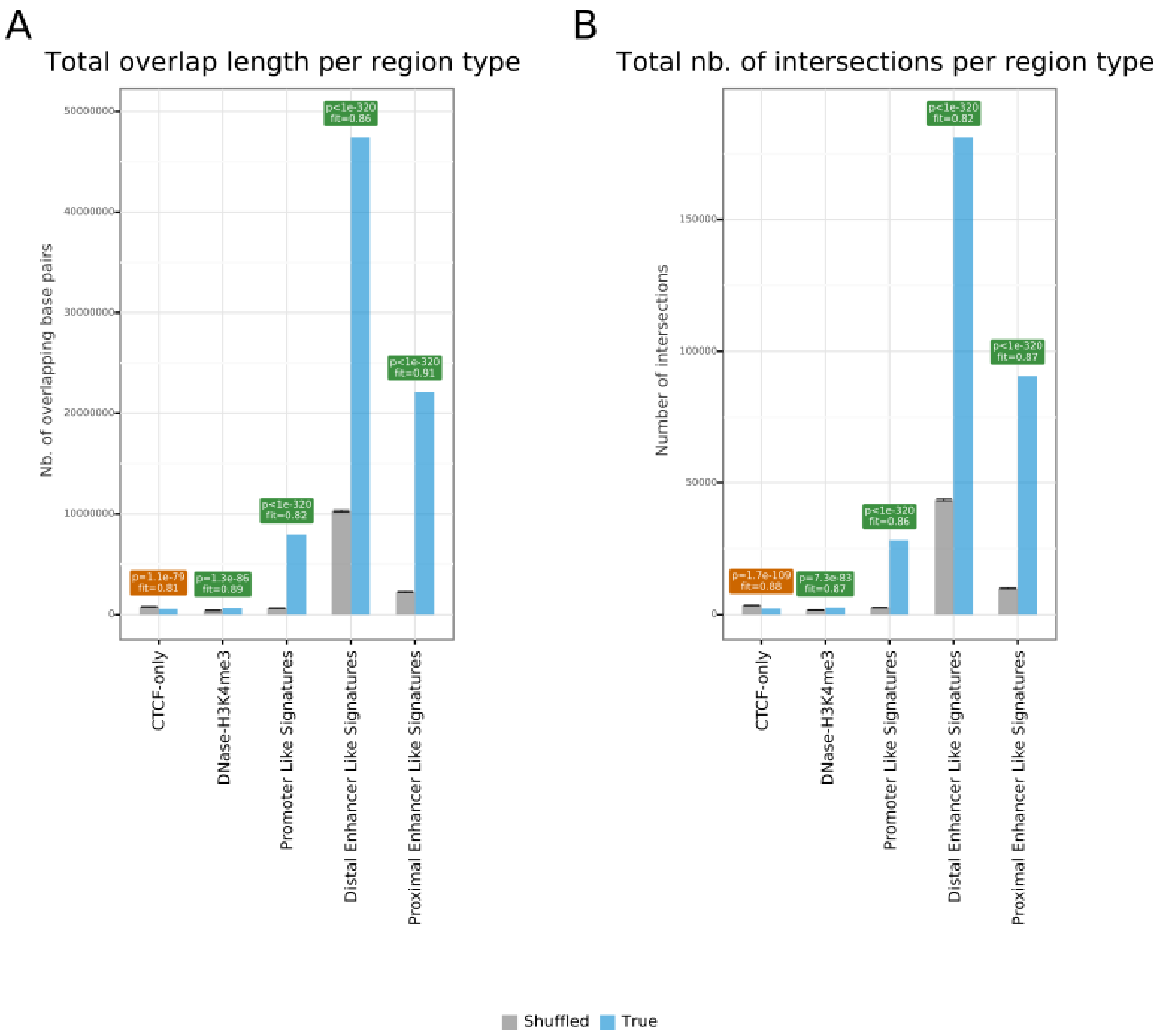

Supplementary Figure 19. **Enrichment analysis for *H. sapiens* CRMs in ENCODE cCREs**. Barplots representing the expected (grey bars) versus observed (blue bars) overlap lengths (**A**) or number of intersections (**B**) between *H. sapiens* CRMs from the robust collection and ENCODE cCREs (x-axis). The plots and computed p-values (green: enrichment; orange: depletion) were obtained using the OLOGRAM command of the GTF toolkit.

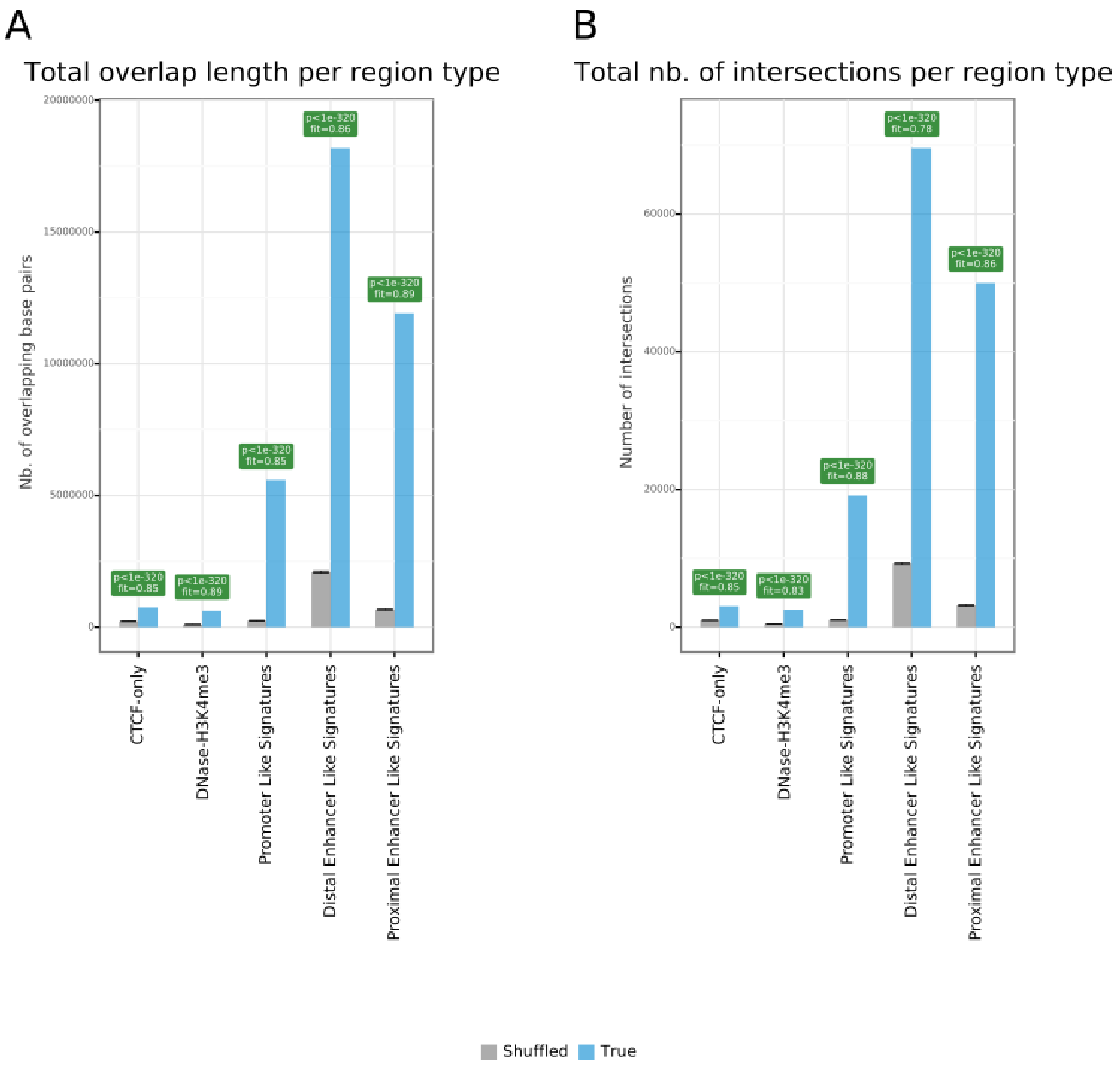

Supplementary Figure 20. **Enrichment analysis for *M. musculus* CRMs in ENCODE cCREs**. Barplots representing the expected (grey bars) versus observed (blue bars) overlap lengths (**A**) or number of intersections (**B**) between *M. musculus* CRMs from the robust collection and ENCODE cCREs (x-axis). The plots and computed p-values (green: enrichment; orange: depletion) were obtained using the OLOGRAM command of the GTF toolkit.

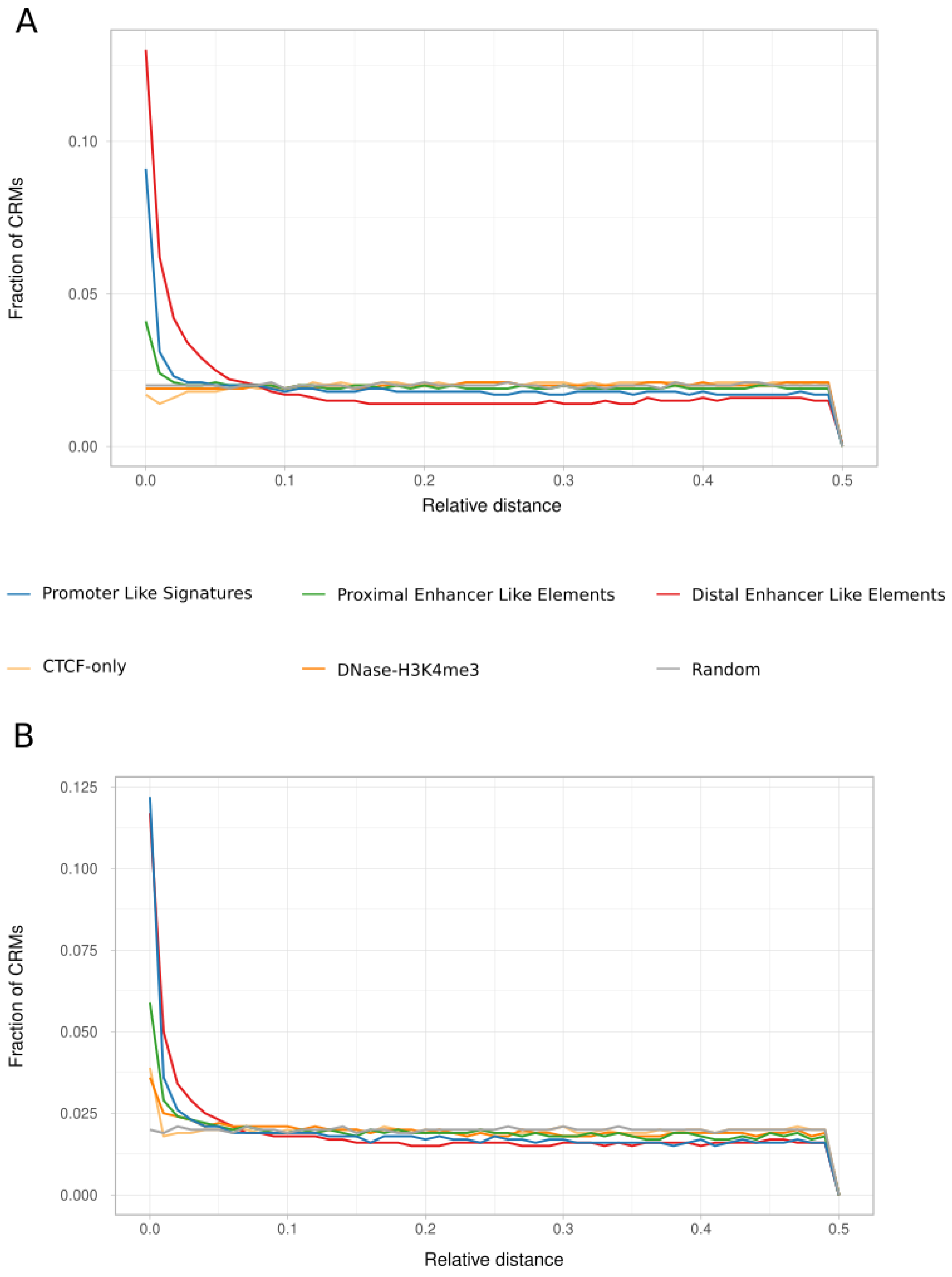

Supplementary Figure 21. Relative distance distributions between CRMs and ENCODE cCREs. Fraction of CRMs in the UniBind robust collection (y-axis) with respect to increasing relative distances (x-axis) from ENCODE cCREs computed using the *bedtools reldist* command for human (**A**) and mouse (**B**). When two genomic tracks are not spatially related, one expects the fraction of relative distance distribution to be uniform.

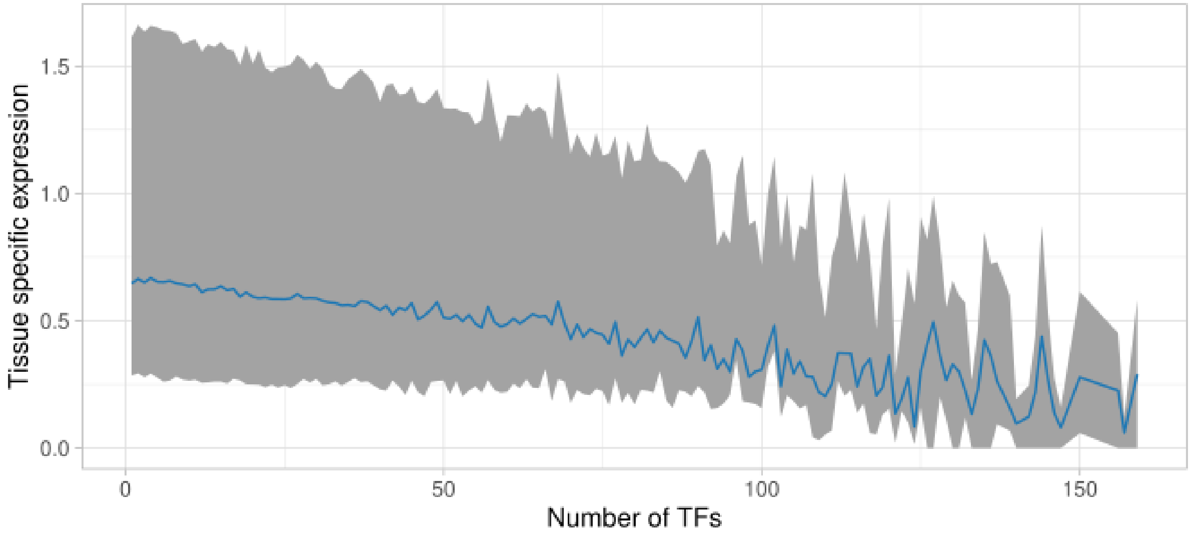

Supplementary Figure 22. **Correlation between enhancer activity and TF binding**. For each enhancer predicted using Cap Analysis of Gene Expression (CAGE) by the FANTOM5 consortium, we computed the number of TFs with overlapping TFBSs in the robust collection of UniBind (x-axis). The figure provides, for each value of the number of TFs found to bind in enhancers, the median (blue line) together with the 10th to 90th percentiles (grey area) of tissue specific activity of these enhancers. The expression measures were derived from CAGE (capturing enhancer RNA expression). The specificity of activity (y-axis) is provided within the [0; 1] range with 0 representing ubiquitous enhancer activity and 1 exclusive expression activity.
